# Pigment Epithelium Derived Factor Peptide Protects Murine Hepatocytes from Carbon Tetrachloride-Induced Injury

**DOI:** 10.1371/journal.pone.0157647

**Published:** 2016-07-06

**Authors:** Shou-Chuan Shih, Tsung-Chuan Ho, Show-Li Chen, Yeou-Ping Tsao

**Affiliations:** 1 Mackay Medical College, New Taipei City, Taiwan; 2 Department of Internal Medicine, Mackay Memorial Hospital, Taipei, Taiwan; 3 Department of Medical Research, Mackay Memorial Hospital, Taipei, Taiwan; 4 Department of Ophthalmology, Mackay Memorial Hospital, Taipei, Taiwan; 5 Department of Microbiology, School of Medicine, National Taiwan University, Taipei, Taiwan; CIMA. University of Navarra, SPAIN

## Abstract

Fibrogenesis is induced by repeated injury to the liver and reactive regeneration and leads eventually to liver cirrhosis. Pigment epithelium derived factor (PEDF) has been shown to prevent liver fibrosis induced by carbon tetrachloride (CCl_4_). A 44 amino acid domain of PEDF (44-mer) was found to have a protective effect against various insults to several cell types. In this study, we investigated the capability of synthetic 44-mer to protect against liver injury in mice and in primary cultured hepatocytes. Acute liver injury, induced by CCl_4_, was evident from histological changes, such as cell necrosis, inflammation and apoptosis, and a concomitant reduction of glutathione (GSH) and GSH redox enzyme activities in the liver. Intraperitoneal injection of the 44-mer into CCl_4_-treated mice abolished the induction of AST and ALT and markedly reduced histological signs of liver injury. The 44-mer treatment can reduce hepatic oxidative stress as evident from lower levels of lipid hydroperoxide, and higher levels of GSH. CCl_4_ caused a reduction of Bcl-xL, PEDF and PPARγ, which was markedly restored by the 44-mer treatment. Consequently, the 44-mer suppressed liver fibrosis induced by repeated CCl_4_ injury. Furthermore, our observations in primary culture of rat hepatocytes showed that PEDF and the 44-mer protected primary rat hepatocytes against apoptosis induced by serum deprivation and TGF-β1. PEDF/44-mer induced cell protective STAT3 phosphorylation. Pharmacological STAT3 inhibition prevented the antiapoptotic action of PEDF/44-mer. Among several PEDF receptor candidates that may be responsible for hepatocyte protection, we demonstrated that PNPLA2 was essential for PEDF/44-mer-mediated STAT3 phosphorylation and antiapoptotic activity by using siRNA to selectively knockdown PNPLA2. In conclusion, the PEDF 44-mer protects hepatocytes from single and repeated CCl_4_ injury. This protective effect may stem from strengthening the counter oxidative stress capacity and induction of hepatoprotective factors.

## Introduction

With its major function of detoxification, the liver is constantly exposed to toxic chemicals from the environment, food and medicines. Moreover, liver injury from viral infections, in the form of acute and chronic hepatitis, is not uncommon. Liver cells also may be damaged by ischemia-reperfusion injury during surgery. Under these circumstances, oxidative stress may lead to liver cell death [1–3]. Liver cell death is followed by an inflammatory response to remove the necrotic tissue. The accumulation and activation of inflammatory cells generates further oxidative stress and lead to more extensive damage [3]. There are intrinsic anti-oxidative mechanisms in the liver, such as superoxide dismutases (SOD), catalases, glutathione peroxidase (GPx), glutathione reductase (GR), peroxiredoxins, thioredoxin, glutathione (GSH) and thiol-containing proteins, which cope with oxidative stress, but the damage and inflammation may exceed their capacity [4]. Strengthening the ability of hepatocytes to handle oxidative stress may, therefore, be of therapeutic value. The involvement and pathophysiology of oxidative stress in liver disease has been studied widely using CCl_4_-induced liver injury. CCl_4_ is an industrial solvent and known hepatotoxin which is metabolically activated by the hepatic microsomal cytochrome p450. This renders it capable of inducing lipid peroxidation of unsaturated fatty acid membranes and organelle membranes, eventually leading to liver cell necrosis. CCl_4_-induced liver injury is used widely in experimental approaches to identify agents that can enhance the capacity of liver cells to handle oxidative stress and thus to protect these cells [5].

Pigment epithelium-derived factor (PEDF) is a 50 kDa secreted glycoprotein produced by the liver. Serum PEDF levels decrease in patients with liver cirrhosis [6]. Previously, we reported that PEDF is an intrinsic anti-fibrotic factor [7]. Hepatic PEDF synthesis decreased dramatically in the liver following CCl_4_ administration but over-expression of PEDF via a viral vector halted the progression of liver fibrosis in an experimental animal [7]. The observation that PEDF prevents the activation of cultured hepatic stellate cells (HSCs), the major cell type involved in liver fibrosis, indicates that this is the mechanism through which PEDF prevents liver fibrosis [7]. PEDF is a protein with multiple biological functions and these functions are carried out by the various functional domains of PEDF. A human PEDF 34-mer (amino acid positions Asp44-Asn77) was originally investigated for the antiangiogenic activities of PEDF [8]. Recently, we performed direct intraperitoneal injection of a synthetic 34-mer peptide to efficiently ameliorate CCl_4_-induced liver fibrosis in mice [9]. In cell culture, the 34-mer suppresses the activation of HSCs [9]. Therefore, the 34-mer seems to encompass the functional domain of PEDF responsible for its anti-fibrotic activity. PEDF contains another well-studied 44 amino acid domain, spanning amino acids Val78-Thr121 of human PEDF. The 44-mer is conserved between the human, mouse and rat with 89% identity and 97% sequence similarity. This 44-mer has neurotrophic and neuroprotective activities but has been reported to lack significant antiangiogenic activity on CNV lesions [8,10] Our previous studies showed that this 44-mer cannot suppress HSC activation in culture [9].

PEDF has been shown to be protective in models of hydrogen peroxide-induced neuronal cell death, ischemic retinal injury and retinal light damage [11–13]. PEDF has been suggested to have a strong antioxidant effect [11,13,14]. There is evidence that the 44-mer domain of PEDF may be responsible for these neuro-protective functions. For example, direct treatment with the 44-mer protects neurons and muscle fibers from injury [15,16]. In addition, it has been found that the 44-mer protects myocardial cells against the reactive oxygen species (ROS) induced by hypoxia [14]. These protective effects against oxidative stress are not seen with 34-mer treatment [14]. Previously, the protective effects of PEDF, particularly the 44-mer peptide of PEDF, were seen only in neurons and muscles. Whether the protective effect of the 44-mer also applies to liver cells is unclear but is potentially important. The present study aimed to address this issue by investigating the hepatoprotective effect of the 44-mer on CCl_4_-treated mice.

## Materials and Methods

### Materials

Recombinant human PEDF derived from stable BHK cell transfectants and preserved in 50 mM sodium phosphate buffer (solvent) was obtained from Chemicon (Temecula, CA). The PEDF peptides 18-mer (Glu97-Ser114), 34-mer (Asp44-Asn77) and 44-mer were synthesized, modified by acetylation at the NH_2_ termini and amidation at the COOH termini for stability, and characterized by mass spectrometry (>90% purity) at GenScript (Piscataway, NJ). PEDF peptides were reconstituted in DMSO (solvent) and used as stock solution (5 mM). PD 98059, GW9662 and STAT3 inhibitor (No. 573096) were purchased from Calbiochem (La Jolla, CA, USA).

### Animal Treatment

Experimental procedures were approved by the Mackay Memorial Hospital Review Board and conducted according to national animal welfare regulations. Acute liver injury was induced in 6-wk-old female C57BL/6 mice (six mice per experimental condition) by a single intraperitoneal injection of CCl_4_ solution (5 ml/kg body weight, as a 1:4 mixture with olive oil). Immediately, the 44-mer peptide and 18-mer control peptide at 10 mg/kg were administered by intraperitoneal injection, twice a day, with an interval of 8 hours.

To induce liver fibrosis, 6-wk-old female C57BL/6 mice (five mice per experimental condition) were injected intraperitoneally twice per week with CCl_4_ solution (5 ml/kg body weight, as a 1:4 mixture with olive oil) or CCl_4_ combined with the 44-mer or 18-mer peptide control peptide at 10 mg/kg for 5 weeks. Mice were injected intraperitoneally twice per week with olive oil as vehicle control. At the indicated time points, mice were anesthetized by an intraperitoneal injection of a mixture of zoletil (6 mg/kg) and xylazine (3 mg/kg), the abdominal cavity was opened, and blood was removed by cardiac puncture from each mouse. Serum and liver specimens were collected for evaluation of the biochemical parameters (AST, ALT), histological and western blot analysis.

### Plasma Studies

Plasma alanine aminotransferase (ALT) and aspartate aminotransferase (AST) activities were determined using a Dri-Chem 4000 analyzer (FUJIFILM, Saitama, Japan) according to the manufacturer’s instructions. Plasma interleukin-1 (IL-1β) and tumor necrosis factor-alpha (TNF-α) levels were measured with the corresponding Mouse ELISA MAX™ Deluxe kits from BioLegend.

### Histopathological examination

The formalin fixed samples were embedded in paraffin and sectioned, and then stained with hematoxylin-eosin for microscopic examination (Nikon, Eclipse, TS100, Japan). At least three different sections were examined per liver sample. The necrotic area was quantified from the photographs using a computer-assisted image analyzer (Adobe Photoshop CS3 10.0). The percentage area of necrosis was determined by dividing the sum area of necrosis by the sum of the reference area of ten fields.

### Measurements of GSH levels, GR activity and GPx activity

Approximately 20% liver homogenate was used to determine the levels of GSH as well as the activities of GR and GPx in liver tissues. The GSH levels were quantified using a Glutathione Assay Kit (BioVision Research Products, Mountain View, CA) according to the manufacturer's recommendations. In brief, the perchloric acid treated homogenate was incubated with the OPA (o-phthalaldehyde) probe and GSH buffer for 40 min at room temperature. The fluorescence was read at 340 nm for excitation and 420 nm for emission on a SPECTRAmax GEMINI XS fluorescence microplate spectrophotometer (Molecular Devices, Sunnyvale, CA). GPx activity and GR activity were measured in liver tissues using commercial assay kits (Cayman Chemical, Ann Arbor, MI) according to the instruction manuals. The protein concentrations of the homogenate were determined by the bicinchoninic acid (BCA) protein assay kit (Pierce).

### TBARS Assay

Malondialdehyde (MDA) was determined using a TBARS (Thiobarbituric acid reactive substances) Assay Kit (Cayman Chemical, Ann Arbor, MI) according to the manufacturer's recommendations. The absorbance was read at 540 nm in a spectrophotometer (Spectra MAX 190, Molecular Devices).

### Sirius red staining

Deparaffinized liver tissue sections were stained for 1 h in 0.1% (w/v) Sirius red (Sigma-Aldrich) in a saturated aqueous solution of picric acid and then rinsed for 30 min in 0.01 N acetic acid to remove unbound dye. For semi-quantitative analysis of liver fibrosis, 10 fields from each slide were randomly selected under a light microscope and the red-stained area per total area (mm^2^/mm^2^) was measured using the Image-Pro Plus 4.5.1 system.

### Immunohistochemistry

Formalin-fixed, paraffin-embedded liver specimens were deparaffinized in xylene and rehydrated in a graded series of ethanol concentrations. Slides were blocked with 10% goat serum for 60 min and then incubated with primary antibody against mouse Bcl-xL (1:100 dilution overnight at 4°C). The slides were subsequently incubated with the appropriate peroxidase-labeled goat immunoglobulin (1:500 dilution; Chemicon, Temecula, CA) for 20 min and then incubated with chromogen substrate (3,3`-diaminobenzidine) for 2 min before counterstaining with hematoxylin.

### Evaluation of apoptosis in vivo

To identify apoptosis of hepatocytes *in vivo*, immunofluorescence staining of apoptotic cells was performed using a TdT-mediated dUTP nick-end labeling (TUNEL)–based kit (Roche Molecular Biochemicals, Indianapolis, IN) according to the manufacturer's instructions. Hepatocyte staining was carried out using primary antibody against albumin (1:150 dilution; Santa Cruz Biotechnology, CA) at 37°C for 2 h, followed by incubation with rhodamine goat IgG (1:500 dilution) for 1 h at room temperature. Nuclei were located by counterstaining with Hoechst 33258 for 7 min. Sections were observed under a Zeiss epifluorescence microscope with a CCD camera (× 400, 10 fields/sample).

### Hepatocyte isolation, cell culture and treatment

Hepatocytes were isolated from the livers of male Sprague-Dawley rats (300–450 g) according to the two step perfusion method, as described previously [7, 17]. Cell viability was determined by trypan blue exclusion and was typically 85–90%. Isolated hepatocytes were resuspended in William’s E medium (WEM; Life Technologies, Inc., Grand Island, NY) supplemented with 10% FBS, 26 mM sodium bicarbonate, 23 mM Hepes, 0.01 U/ml insulin, 2 mM L-glutamine, 10 nM dexamethasone, 5.5 mM glucose, 100 U/ml penicillin, and 100 U/ml streptomycin and plated at a concentration of 5 × 10^4^ cells/cm^2^ in collagen coated plates (Corning Costar Corporation, Cambridge, Mass.). Cell incubations were performed in a humidified incubator at 5% CO_2_ and 37°C temperature. After plating for 6 h, non-adherent cells and debris were removed by washing with 10% FBS-WEM. After 18 h, the cells were shifted to a serum-free medium, consisting of WEM supplemented with 2 mM glutamine, 0.01 U/ml insulin, 10 μM hydrocortisone, PEDF (10 nM), PEDF-derived peptides (10 μM, unless differently specified), 1mM NAC or 10 ng/ml TGF-β1 (R & D Systems, Minneapolis, MN) for 24 h.

### Cell culture of HCC cell lines

Human hepatoma HepG2 and HuH7 cells were from the American Type Culture Collection (ATCC, Manassas, VA, USA) and grown in DMEM supplemented with 10% FBS, 50 U/ml penicillin and 50 μg/ml streptomycin. Treatments with PEDF and peptides were performed on cells (5 × 10^4^ cells/cm^2^) seeded in serum free DMEM medium.

### Quantitative real-time RT-PCR

The total RNA was extracted from cells using TRIzol (Invitrogen) and treated with RNase-free DNase I (Qiagen, Santa Clarita, CA) to remove genomic DNA and then purified with an RNA purification kit (Dynabeads; Invitrogen). Synthesis of cDNA was performed by Superscript III (Invitrogen). Quantitative real-time PCR was performed in a GeneAmp 7700 sequence detection system (Applied Biosystems, Foster City, CA). Amplification was carried out in a total volume of 40 μl containing 3 pmol of primers, serially diluted RT product and SYBR Green PCR core reagents (Applied Biosystems). Primers used in the experiment were listed in S1 Table. The step-cycle program was set for denaturing at 95°C for 15 s, and annealing and extension at 58°C for 1 min, for a total of 40 cycles. All determinations were measured in triplicate. The cycle threshold (*Ct*) values corresponded to the PCR cycle number at which fluorescence emission in real time reaches a threshold above the base-line emission were analyzed using GeneAmp 7700 SDS software. The *Ct* value of the PCR product of interest and a control mRNA (GAPDH) were then used to calculate relative quantities of mRNA between samples.

### RNA interference

Rat siRNA (GenDiscovery Biotechnology, Inc, Taiwan) used in the experiment contained a pool of three individual siRNA sequences for the target gene and was listed in S2 Table. The siRNA was resuspend with 1000 μl of 1× siRNA buffer (Cat #B-002000-UB-100; Dharmacon) to yield a final concentration of 20 μM, aliquot siRNA and store at -20°C until use. Nonsilencing control siRNAs (sc-37007 and sc-44230) was purchased from Santa Cruz Biotechnology. For the transfection procedure, hepatocytes were grown to 70% confluence and then placed in WEM supplemented with Glutamax. siRNA was transfected using Lipofectamine™ 2000 reagent (Invitrogen, Carlsbad, CA) according to the manufacturer’s instructions. The final concentration of siRNA was 50 nM. At 24 h after siRNA transfection, hepatocytes were resuspended in new culture media for recovery for 24 h, and then treated with PEDF or the 44-mer.

### Western blot analysis

Cells were scraped into lysis buffer (150 μL/35-mm well) containing 20 mM HEPES (pH 7.4), 1% SDS, 150 mM NaCl, 1 mM EGTA, 5 mM β-glycerophosphate, 10 mM sodium pyrophosphate, 10 mM sodium fluoride, 100 μM sodium orthovanadate, 10 μg/mL leupeptin, and 10 μg/mL aprotinin. The lysate was resolved by SDS-PAGE, electrotransferred to polyvinylidene difluoride membranes (Millipore, Bedford, MA), and processed for immunoblot analysis. Antibodies used in this study were against PEDF (1:1000 dilution; MAB1059; Millipore), Bax, type I collagen 1A1, α-SMA, laminin-R, and Bcl-xL (1:1000 dilution; all from Santa Cruz Biotechnology), phospho-Stat3 (Tyr705), LRP6, PPARγ (1:1000 dilution; all from Cell Signaling Technology), PNPLA2/ATGL (1:1000 dilution; GTX59676; GeneTex, Inc) and cleaved caspase 3 (1:500 dilution; Abcam). Proteins of interest were detected using the appropriate IgG-HRP secondary antibody and ECL reagent. X-ray films were scanned on a Model GS-700 Imaging Densitometer (Bio-Rad Laboratories, Hercules, CA) and analyzed using Labworks 4.0 software. Blots from at least three independent experiments were used for quantification.

### Detection of reactive oxygen species (ROS) by H_2_DCFDA

Intracellular ROS generation was assayed using 2`,7`-dichlorodihydrofluorescein diacetate (H_2_DCFDA; Molecular Probes, Eugene, OR) which, when oxidized by ROS, releases the green fluorescent compound 2`,7`-dichlorofluorescein (DCF). To detect ROS by spectrofluorometric assay, 1.2 × 10^4^ cells were seeded in a collagen-coated 96-well plate in WEM supplemented with 10% FBS for 24 h. The cells were then incubated in serum-free media with or without PEDF peptides for an additional 6 h. The hepatocytes were subsequently washed with PBS (pH 7.4) and then incubated with fresh medium containing 5 μM H_2_DCFDA in the dark for 15 min at 37°C. Fluorescence (excitation, 488 nm; emission, 520 nm) was measured with a Spectra MAX GEMINI Reader (Molecular Devices, Sunnyvale, CA, USA). The background fluorescence from control wells without the addition of H_2_DCFDA was subtracted from the experimental readings.

### Statistics

The results are expressed as the mean ± standard error of the mean (SEM). ANOVA was used for statistical comparisons. *P* <0.05 was considered significant.

## Results

### The 44-mer alleviates acute liver injury induced by CCl4

**Fig 1:**
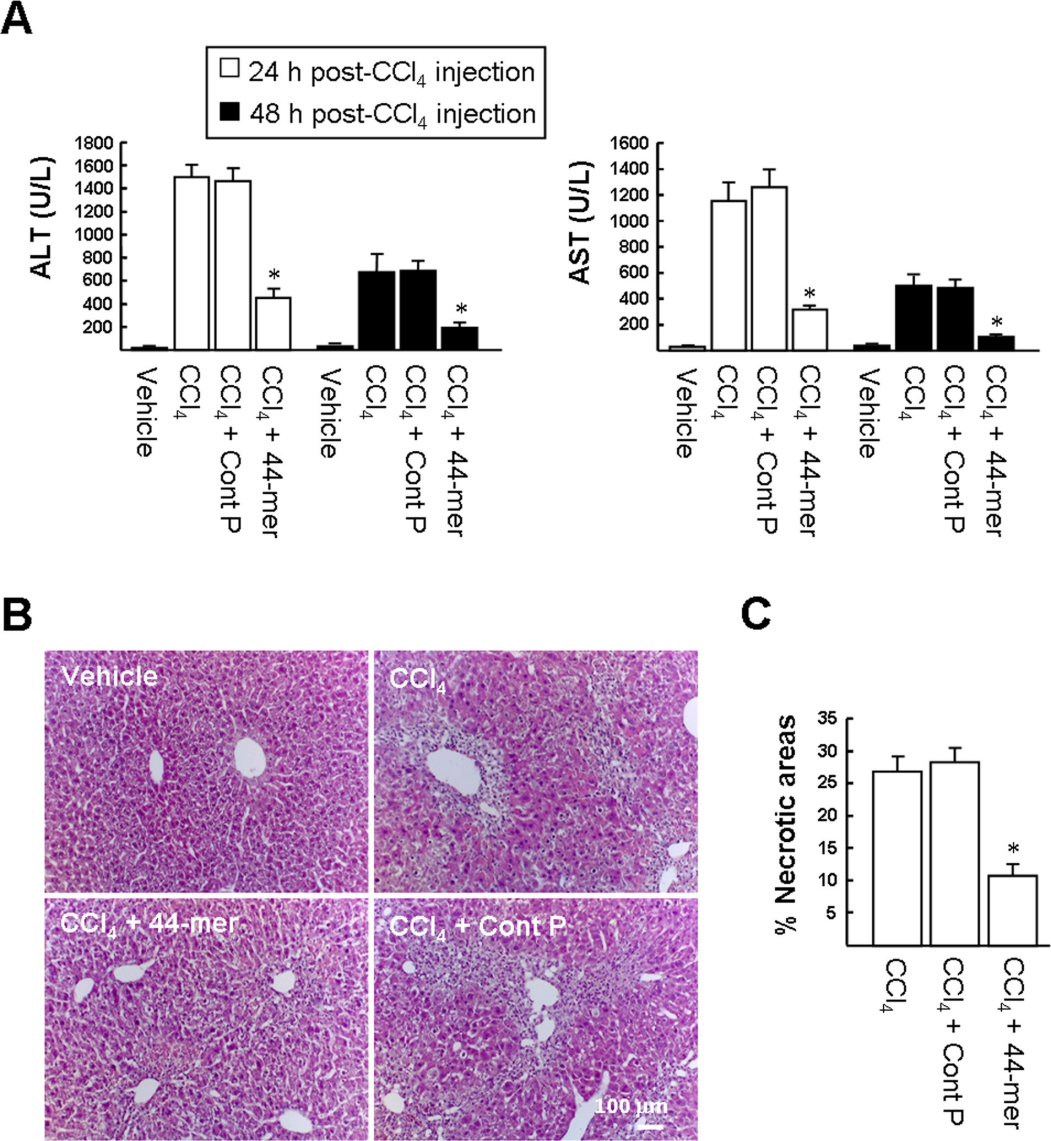
The 44-mer protects the liver against CCl_4_-induced acute liver injury. (A) Levels of liver function markers, serum ALT and AST, on days 1 and 2 after CCl_4_ injection. Vehicle: olive oil-treated mice. Values are expressed as mean ± S.D., *P <0.05 versus CCl_4_+control peptide-treated group. (B) Histological evaluations of liver sections 48 h after CCl_4_ injection. Representative photomicrographs of liver histology, liver sections from mice treated with CCl_4_ alone and in combination with the 44-mer and control peptide stained with hematoxylin and eosin are shown. (C) Percentage of necrotic areas after CCl_4_ treatment. *P <0.001 versus CCl_4_+control peptide-treated group.

To determine whether the 44-mer could prevent liver damage in CCl_4_-intoxicated mice, acute liver injury was induced by a single injection of CCl_4_ solution. Subsequently, the 44-mer peptide and an 18-mer control peptide (Cont P) were administered by intraperitoneal injections twice per day with an interval of 8 hours. Liver damage after CCl_4_ injection was quantified by measuring ALT and AST in plasma (Fig 1A). ALT and AST levels were raised at 24 h and 48 h after the intraperitoneal injection with CCl_4_, compared to mice injected with olive oil vehicle. However, ALT levels raise was markedly prevented in mice treated with CCl_4_/44-mer, compared to the CCl_4_ and CCl_4_/control peptide-treated mice at 24 h (457±77.8 versus 1504±95.1 and 1464±120.8) and 48 h (186±36.3 versus 670±156.7 and 678±92.5). AST levels raise was also markedly prevented in mice treated with CCl_4_/44-mer, compared to the CCl_4_ and CCl_4_/control peptide-treated mice at 24 h (315±31.5 versus 1154±145.5 and 1262±138.5) and 48 h (104±9.6 versus 500±74.4 and 477±66.1). In addition, the 44-mer peptide introduced into mice in doses ranging from 5 ~ 20 mg per kg of mouse body weight have been used to test their effects on the elevated plasma AST/ALT levels at 24 h and 48 h after single injection of CCl_4_ solution. The results revealed that the 44-mer dosages including 7.5, 10 and 20 mg displayed a similar ability to suppress the plasma AST and ALT levels increased at 24 h and 48 h (S1 Fig). The attenuation of the increases of plasma AST and ALT indicated that the 44-mer has a protective effect on hepatocytes.

Liver sections from the mice 48 h after CCl_4_ injection were examined for the effect of the 44-mer treatment by H&E staining (Fig 1B). The livers of the CCl_4_-intoxicated mice showed gross necrotic lesions, loss of cellular boundaries and broad infiltration of inflammatory cells around the portal areas. CCl_4_-intoxicated mice treated with the 44-mer had significantly smaller areas of necrosis than the CCl_4_ and CCl_4_/control peptide groups (10.9 ± 1.7% *versus* 26.8 ± 2.5% and 28.3 ± 2.4%, respectively). This result supports the conclusion that the 44-mer suppresses elevation of serum AST and ALT in CCl_4_-intoxicated mice.

### The 44-mer treatment suppresses lipid peroxidation induced by CCl_4_

Lipid peroxidation is formed by free radicals of CCl_4_ metabolism and plays a vital role in the CCl_4_-induced liver damage [5]. We monitored the levels of malondialdehyde (MDA; a lipid peroxidative product of cell membranes) by TBARS measurement. As shown in Table 1, CCl_4_ treatment significantly increased the hepatic MDA level compared to the vehicle control group (3.73±0.36 versus 1.55±0.23 nmol/mg liver protein). Notably, administration of the 44-mer markedly decreased the MDA levels by 2.51±0.17 nmol/mg. Control peptide did not prevent the increase of MDA in mouse livers damaged by CCl_4_. In evaluating the anti-oxidative status of the liver in experimental animals, the total hepatic GSH levels of the CCl_4_-treated group were found to be markedly depleted compared to those of the vehicle control mice (19.5±1.50 versus 32.5±2.74 μg/mg), whereas the 44-mer treatment significantly increased the GSH levels up to 29.0±1.79 μg/mg. In addition, glutathione reductase (GR) and glutathione peroxidase (GPx) activities were found to be decreased significantly in CCl_4_-intoxicated animals, compared to the vehicle group (Table 1). However, mice treated with CCl_4_/44-mer showed significant increases in GR and GPx compared to the CCl_4_/Cont P-treated group (**P <0.05). Collectively, the reduced MDA generation and elevated GSH level in the 44-mer/CCl_4_ group indicated that the 44-mer was antioxidative and beneficial to the recovery of the liver from acute injury.

**Table 1:** Effect of the 44-mer on assessment of oxidative stress.

Group	MDA	GSH	GR	GPx
Vehicle	1.55±0.23	32.5±2.74	6.90±1.09	4.48±0.82
44-mer	1.49±0.09	34.6±2.31	7.08±0.83	4.52±0.81
CCl_4_	3.73±0.36*	19.5±1.49*	4.04±0.51*	2.56±0.69*
CCl_4_+44-mer	2.51±0.17**	29.0±1.79**	5.36±0.56**	3.46±0.81**
CCl_4_+Cont P	3.63±0.27	19.0±1.86	4.02±0.67	2.74±0.64

The values presented are the means ± standard error of the mean (n = 6).

* *P*<0.05 compared with the vehicle group

** *P*<0.05 compared with the CCl_4_/Cont P-treated group

### The 44-mer suppresses the CCl_4_-induced inflammatory response

**Fig 2:**
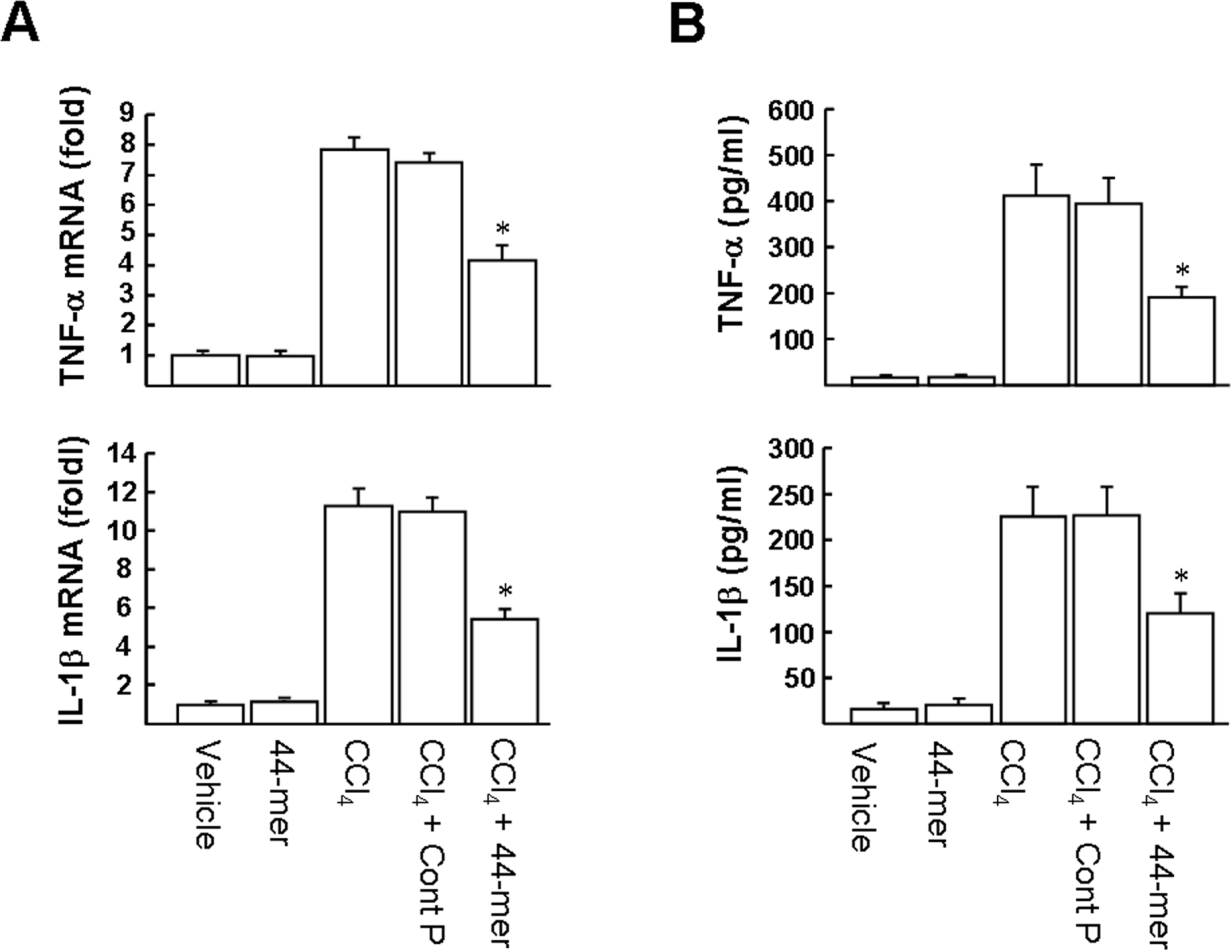
Effects of the 44-mer on CCl_4_-induced liver inflammation. (A) Mice were treated with CCl_4_ for 12 h. Liver tissues were then harvested and assayed by quantitative real-time PCR. (B) Serum levels of cytokines. Values are expressed as mean ± SE in each group. *P <0.05 versus CCl_4_+control peptide-treated group.

It has been suggested that inflammation may increase hepatic injury in CCl_4_-induced serial pathological damage [18–20]. Among the proinflammatory mediators, it was reported that the liver injury induced by CCl_4_ was ameliorated in mice pretreated with anti-TNF-α antibody or soluble TNF receptor [19,20]. As shown in Fig 2A, after CCl_4_ treatment for 12 h, the mRNA levels of proinflammatory mediators, including TNF-α and IL-1β, were significantly up-regulated by 7.8 and 11.3 fold, respectively, compared to the vehicle group. However, administration of the 44-mer apparently repressed the mRNA expression of hepatic TNF-α, and IL-1β by factors of 1.9-fold and 2.1-fold, respectively, compared to the CCl_4_/Cont P-treated group. Next, we examined the plasma levels of proinflammatory cytokines. As shown in Fig 2B, TNF-α and IL-1β were dramatically increased in plasma 24 h after CCl_4_ administration, compared to the vehicle group. Administration of the 44-mer markedly decreased the levels of inflammatory mediators induced by CCl_4_ (TNF-α: 190.2±23.7 versus 411.8±64.6 pg/ml and IL-1β:119.6±21.5 versus 225.0±31.6 pg/ml). Taken together, our results indicate that the 44-mer attenuated CCl_4_-induced inflammatory responses in mice.

### The 44-mer protects hepatocytes against CCl_4_-induced apoptosis

**Fig 3:**
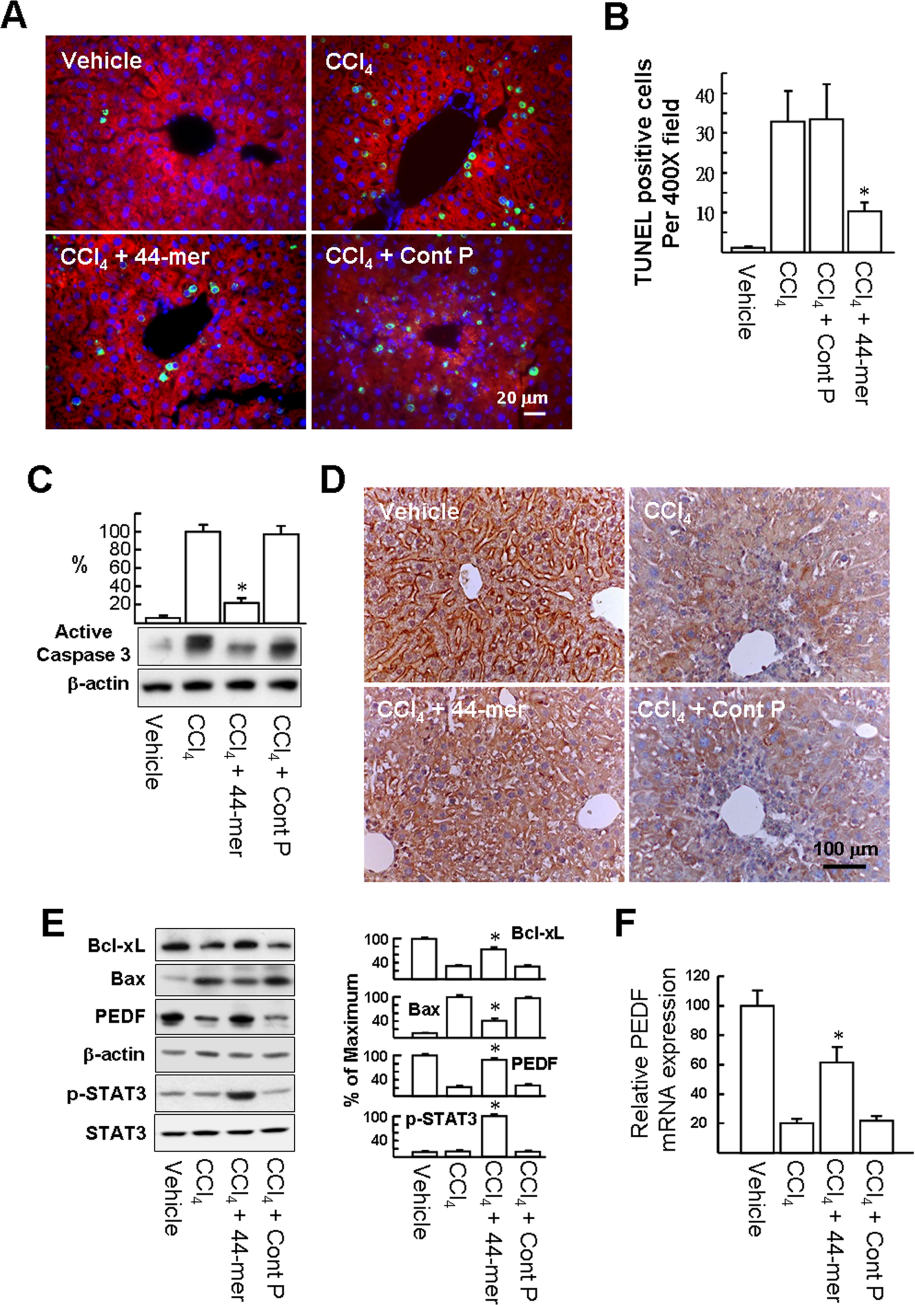
The 44-mer suppresses hepatocyte apoptosis in CCl_4_-treated mice. Apoptosis of hepatocytes was quantified 24 h after administration of a single dose of CCl_4_ or CCl_4_+44-mer. (A) Liver sections were double-stained with TUNEL, to identify apoptotic cells (*green*), and for albumin, to identify hepatocytes (*red*). (B) The number of TUNEL-positive cells was determined as the mean of 5 different areas at ×400 magnification of each section. *P <0.05 *versus* CCl_4_+control peptide-treated group. (C) Western blot analysis of active caspase-3 protein in response to CCl_4_ and CCl_4_+ 44-mer and control peptides. Representative blots and densitometric analysis are shown from 3 independent experiments. *P <0.05 versus CCl_4_+control peptide -treated group. The 44-mer partially sustains hepatic Bcl-xL protein levels in mice for 24 h, following a single injection of CCl_4_. (D) Liver sections were immunostained with anti-Bcl-xL antibody and counterstained with hematoxylin. Secondary antibody only was used as negative control. (E) Liver protein extracts were harvested and subjected to western blot analysis with antibodies as indicated. Representative blots and densitometric analysis from 3 independent experiments are shown. The immunoblots were scanned and quantitated at individual sites and normalized to β-actin. *P <0.02 versus CCl_4_+control peptide -treated group. (F) The Effect of CCl_4_, 44-mer peptide, and their combination on the relative mRNA levels of PEDF in mouse liver. Results represent the mean ± SE from three independent experiments. *P <0.05 versus CCl_4_+control peptide -treated group.

Apoptosis of hepatocytes is one of the recognized features of liver damage induced by CCl_4_ and the maximum number of apoptotic hepatocytes reportedly may be observed at 24 ~ 48 h after a single administration of CCl_4_ [21,22]. We analyzed the changes in cell apoptosis using TUNEL analysis (*green color*; Fig 3A). Liver sections were further immunostained for the hepatocyte marker, albumin (*red color*). Numerous TUNEL-positive hepatocytes, concentrated around portal areas, were identified in the CCl_4_-treated mice and CCl_4_/Cont P-treated mice, compared to the vehicle-treated group (cell numbers per 400× field: 32.7±7.8 and 33.1± 8.9 *versus* 1.0±0.37; Fig 3B), whereas the number of TUNEL-positive hepatocytes was reduced markedly in the presence of the 44-mer (10.2±2.2). We also evaluated the levels of active caspase 3 in liver protein extracts harvested one day after CCl_4_ injection (Fig 3C), when the levels of active caspase 3 were reduced in the group treated with the 44-mer by a factor of 4.5-fold, compared to the CCl_4_/Cont P group. Taken together, these data indicate that the 44-mer inhibits apoptosis of hepatocytes induced by CCl_4_.

### The 44-mer prevents hepatic Bcl-xL and PEDF down-regulation induced by CCl_4_

Hepatocyte-specific Bcl-xL-deficient mice display continuous apoptosis of hepatocytes, indicating the crucial role of this protein in determining hepatocyte survival or death [23]. Because CCl_4_ also suppresses Bcl-xL expression in the mouse liver [24], we investigated whether the 44-mer could maintain Bcl-xL protein expression in CCl_4_ intoxicated livers. Immunohistochemical staining with anti-Bcl-xL antibody showed that the Bcl-xL signal was clearly stronger in normal liver than in livers subjected to CCl_4_ administration (Fig 3D). However, 44-mer treatment partially sustained the Bcl-xL level compared to mice treated with CCl_4_ alone. Next, we investigated expression of hepatoprotective factors, including Bcl-xL, PEDF and phosphor-STAT3, by western blot analysis. As shown in Fig 3E, at 48 h after the intraperitoneal injection of CCl_4_, the amounts of hepatic Bcl-xL and PEDF were markedly reduced compared to the vehicle control (32.6±2.4% and 22.4±3.3% versus 100%), whereas Bax (an inducer of apoptosis) was increased by 9.6-fold. However, the Bcl-xL and PEDF levels were partially sustained by treatment with the 44-mer (74.0±5.2% and 89.8±5.1%), compared to the vehicle control. The 44-mer treatment also suppressed the hepatic Bax levels compared to CCl_4_-treated mice (41.8±5.6% versus 100%). Moreover, 44-mer administration resulted in an 8-fold induction of the phosphor-STAT3 protein compared to CCl_4_/Cont P-treated mice. In addition, PPARγ levels declined along with the reduction of PEDF when mice were exposed to a single intraperitoneal CCl_4_ injection for 48 h as our previously reported [7]. However, the alteration of PPARγ levels was partially blocked by treatment with the 44-mer (24.6% versus 86.3%), compared to the basal levels (vehicle set as 100%; S2 Fig), indicating that CCl_4_-induced decrease of PPARγ protein levels is prevented by 44-mer treatment. The PEDF mRNA levels were also partially sustained by treatment with the 44-mer, compared to the vehicle control (61.3% versus 22.0%; Fig 3F). Collectively, the 44-mer ameliorates CCl_4_-induced down-regulation of hepatic Bcl-xL, PPARγ and PEDF, as well as inducing STAT3 activation.

### Exogenous PEDF peptide increases PEDF gene expression in hepatocytes and hepatocellular carcinoma cells

**Fig 4:**
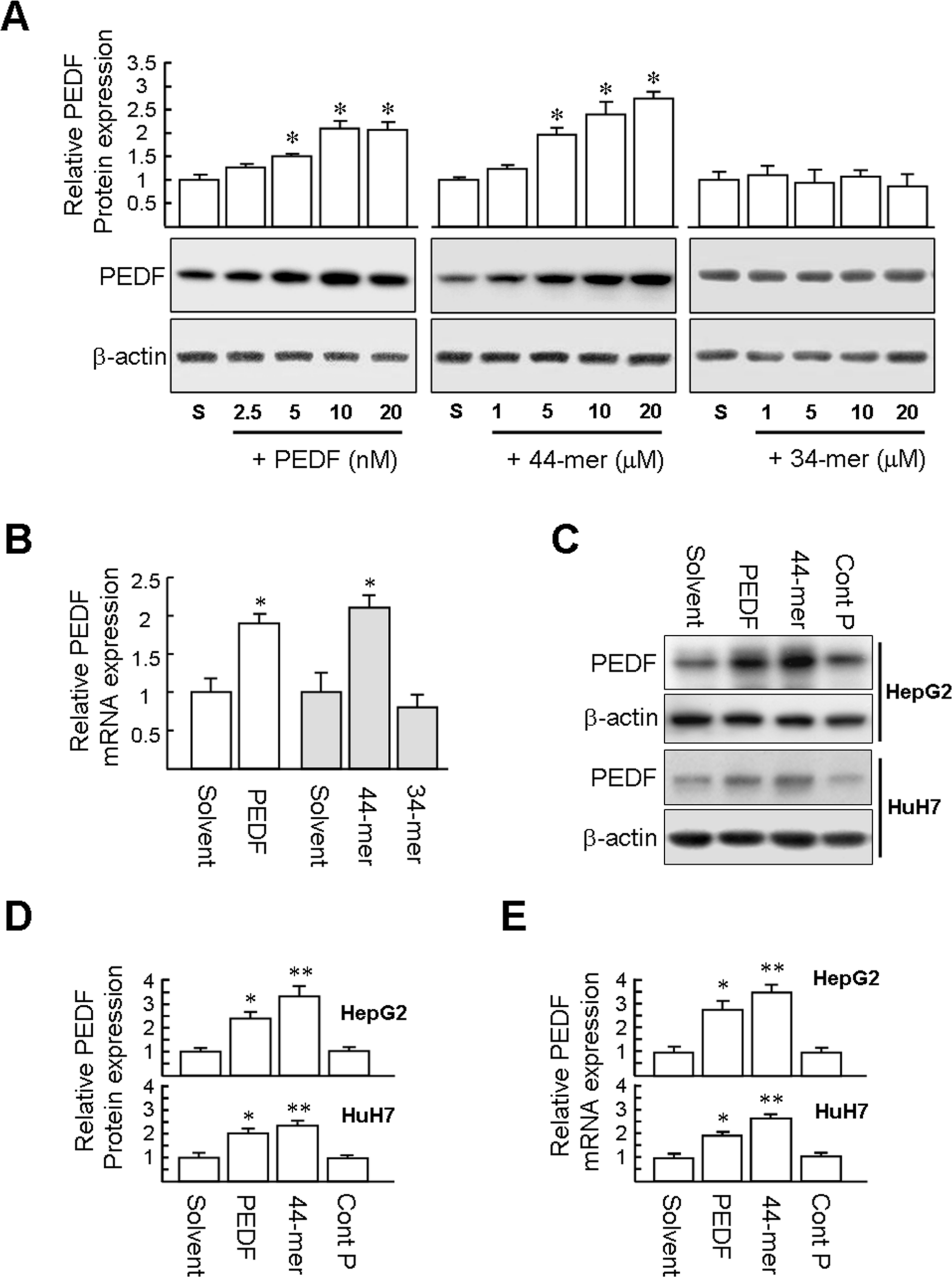
PEDF and the 44-mer induce endogenous PEDF expression in primary rat hepatocytes and human hepatoma cells. (A) Western blot analysis. Primary rat hepatocytes were treated with varying doses of PEDF, 44-mer or 34-mer peptide for 24 h. Protein expression were calculated as the fold increase of PEDF expression compared to the solvent (S) controls. Representative immunoblots and densitometric analysis are shown as the mean ± SE of 3 independent experiments. *P<0.05 versus solvent-treated cells. (B) Relative mRNA levels of PEDF. Primary rat hepatocytes were treated with 10 nM PEDF, 10 μM 44-mer or 34-mer peptide for 24 h. PEDF mRNA was estimated by quantitative real-time RT-PCR. Results represent the mean ± SE from three independent experiments, each performed in quadruplicate. *P <0.05 versus solvent-treated cells. (C-E) Human hepatoma HepG2 and HuH7 cells were treated with 10 nM PEDF, 10 μM 44-mer or control peptide for 24 h. Cells were then harvested and assayed by western blot analysis and quantitative real-time PCR. Representative immunoblots and densitometric analysis are shown as the mean ± SE (n = 3). Real-time PCR data are presented as the mean ± SE (n = 4). *P <0.05 versus solvent-treated cells. **P <0.05 versus control peptide-treated cells.

It has been reported that PEDF positively regulates its own expression in cultured Müller cells and in the livers of mice treated repeatedly with CCl_4_ [7,25]. We examined whether PEDF and synthetic PEDF-derived short peptides positively regulates its own expression in primary culture of rat hepatocytes within 24 hours of isolation. We measured the levels of PEDF protein in hepatocytes by western blotting, and results revealed that PEDF treatment (5, 10, and 20 nM; 24 h) increased endogenous PEDF protein levels by ~ 1.5, 2.1, and 2.1-fold, compared with solvent-treated cells (Fig 4A). In addition, the 44-mer treatment (5, 10, and 20 μM; 24 h) increased PEDF protein levels by ~ 2.0, 2.4, and 2.7-fold in a dose-dependent fashion, compared with solvent-treated cells. In contrast, 34-mer had no effect on the PEDF protein level in primary rat hepatocytes. The mRNA level of PEDF was increased either by PEDF (1.9-fold) or the 44-mer (2.1-fold) treatments, respectively, but 34-mer treatment did not increase PEDF gene expression (Fig 4B). Also, we examined the induction of PEDF gene expression by PEDF and the 44-mer in HepG2 and HuH7 cells. Exposure of HepG2 cells to PEDF and the 44-mer for 24 h resulted in a 2.4-fold and 3.3-fold induction of the PEDF protein, compared to PEDF solvent- and control peptide-treated cells, respectively (Fig 4C and 4D). Meanwhile, the mRNA level of PEDF increased in HepG2 cells incubated with PEDF and the 44-mer (2.9-fold and 3.7-fold, respectively; Fig 4E). PEDF and the 44-mer treatment also significantly increased PEDF protein and mRNA levels in HuH7 cells. Collectively, PEDF/44-mer treatment increased the steady-state mRNA and protein levels of PEDF in cultured rat hepatocytes and human hepatocellular carcinoma cells.

### PEDF and the 44-mer protect primary rat hepatocytes against apoptosis induced by serum deprivation and TGF-β1

**Fig 5:**
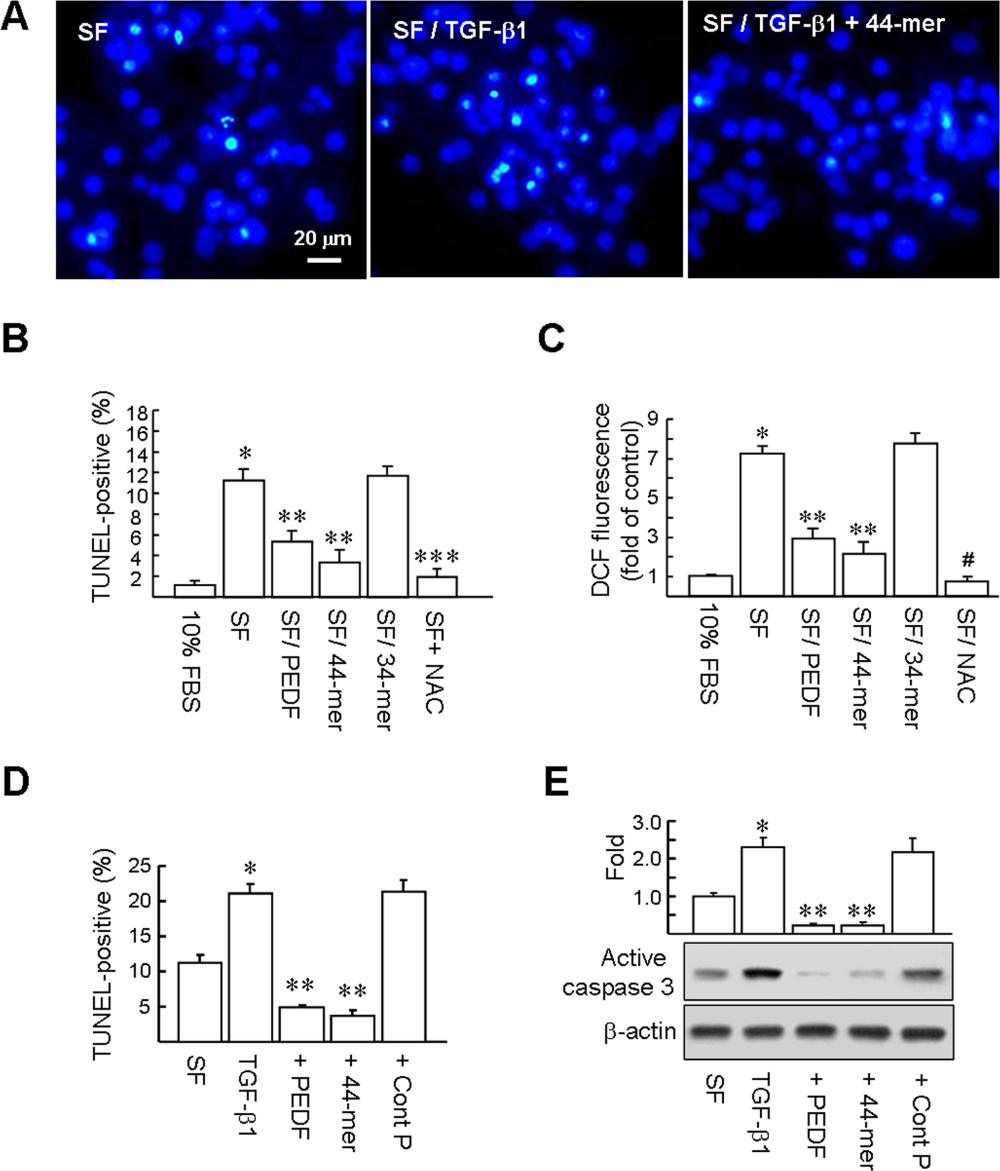
PEDF and the 44-mer protect primary rat hepatocyte against apoptosis induced by serum deprivation and TGF-β1. Hepatocytes were either left cultured in serum-free (SF) medium or treated with PEDF peptides, TGF-β1 or a combination of PEDF peptide and TGF-β1 for 24 h. (A) Apoptosis was determined by TUNEL staining (*green dots*) and doubly stained with Hoechst 33258 (*blue dots*). A representative of four independent experiments is shown. (B) The percentage of cell death was quantified by dividing the number of TUNEL-positive cells to a population of 2000 counted cells per condition. Control: hepatocytes cultured in medium with 10% FBS. Graphs represent means ± SE (n = 4). *P<0.001 versus cell cultured in 10% FBS medium. **P < 0.02 relative to the cells cultured in SF medium. ***P< 0.001 versus cells cultured in SF medium. (C) Analysis of ROS generation induced by serum deprivation. Hepatocytes were either left cultured in SF medium or treated with PEDF peptides or with 1 mM NAC for 24 h. ROS production was expressed as DCF fluorescence intensity and quantified by spectrofluorimetry. *P<0.001 versus cells cultured in 10% FBS medium. **P < 0.02 relative to the cells cultured in SF medium. ^#^*P*< 0.001 versus cells cultured in SF medium. (D) Apoptosis was determined by TUNEL assay and the percentage of cell death was quantified. Graphs represent means ± SE (n = 4). *P<0.001 versus cells cultured in SF medium. **P < 0.05 versus TGF-β1 treated cells. (E) Western blotting analysis of active caspase-3. Representative blots and densitometric analysis are shown. Graphs represent means ± SE (n = 3). *P<0.01 versus cells cultured in SF medium. **P < 0.001 versus TGF-β1 treated cells.

Primary rat hepatocytes are a widely used experimental model to estimate drug toxicity and cell protection. In this regard, It has been showed that withdrawal of serum from the culture medium causes ROS production in, and apoptosis of, primary rat hepatocytes [17]. We tested whether PEDF and the 44-mer could prevent serum deprivation-induced hepatocyte apoptosis in culture. We incubated primary rat hepatocytes with serum-free (SF) medium and examined the TUNEL-positive cells at 24 h (*green fluorescence*; Fig 5A). Quantitatively, greater numbers of hepatocytes cultured in SF medium were TUNEL-positive, compared to cells cultured in 10% FBS medium (11.3±1.1% *versus* 1.2±0.4%; 5B). Importantly, there was a significant reduction of apoptosis of cells treated with PEDF and the 44-mer (5.3±1.1% and 3.3±1.2%, respectively). The 34-mer did not prevent apoptosis induced by serum deprivation. In addition, the effect of PEDF (5 ~ 20 nM) and the 44-mer (5 ~ 20 μM) on serum deprivation–induced hepatocyte apoptosis was dose-dependent (S3 Fig).

To evaluate the effect of serum deprivation on intracellular ROS formation in primary rat hepatocytes, we used H_2_DCFDA, a dye that generates green fluorescence (DCF-fluorescence) when oxidized by ROS. Spectrofluorometric assay revealed that the DCF-fluorescence increased 7.3-fold when hepatocytes were cultured in SF medium for 24 h, compared to hepatocytes cultured in medium containing 10% FBS. However, the DCF-fluorescence level in hepatocytes cultured with PEDF and the 44–mer were reduced by a factor of 2.0-fold and 2.9-fold, respectively, compared to SF alone (Fig 5C). The 34-mer had no such ROS reduction effect. In addition, treatment with antioxidant *N*-acetyl cysteine (NAC; 1 mM) reduced the DCF-fluorescence and cell apoptosis induced by serum deprivation. This indicates that PEDF 44-mer peptide protects hepatocytes through the reduction of ROS.

TGF-β1 is known to be an apoptosis inducer for the rat hepatocytes [26]. We then determined whether PEDF/44-mer could inhibit apoptosis induced by the TGF-β1. We incubated primary rat hepatocytes with SF medium supplemented with TGF-β1 and examined the TUNEL-positive cells at 24 h (Fig 5A and 5D). Quantitatively, greater numbers of hepatocytes cultured in SF/TGF-β1 medium were TUNEL-positive, compared to cells cultured in SF medium (21.0±1.4% *versus* 11.3±1.1%). Addition of PEDF and the 44-mer to the medium significantly decreased TGF-β1-induced apoptosis to 4.8±0.4% and 3.7±0.8%, respectively. The expression of the apoptosis-related proteins, active caspase 3, was detected by western blotting (Fig 5E). Primary rat hepatocytes incubated with SF/TGF-β1 exhibited a 2.3-fold increase in the active caspase 3 level over cells incubated in SF medium at 24 h. PEDF and the 44-mer significantly reduced the active caspase 3 levels compared to cells incubated in SF medium and SF/TGF-β1 medium. In contrast, no inhibitory effect was detected when cells were incubated with control peptide. Collectively, our in vitro results revealed that PEDF and the 44-mer exert a direct protective effect on hepatocytes.

### The STAT3 signaling pathway and PNPLA2 PEDF receptor are responsible for the hepatocyte protection by PEDF

**Fig 6:**
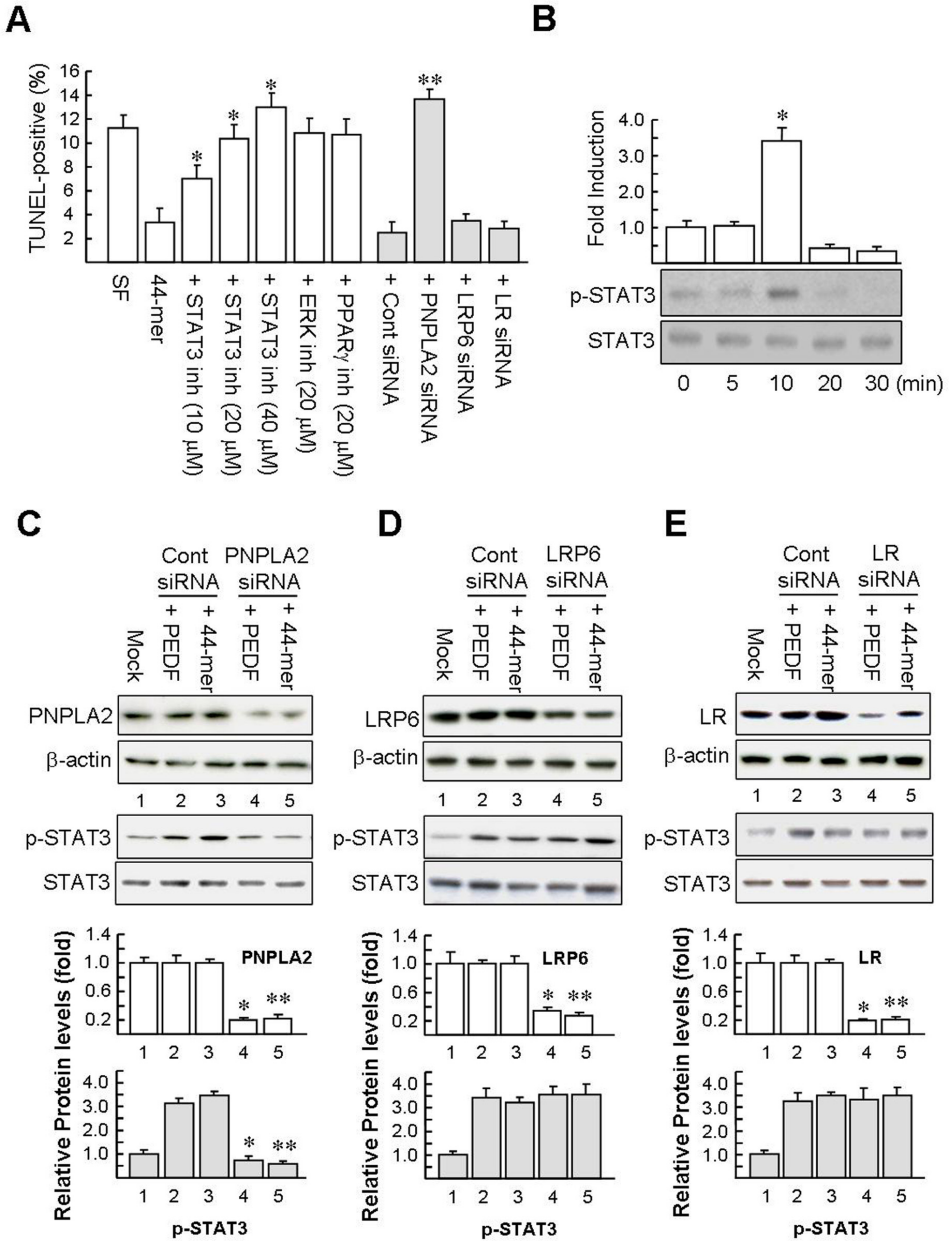
Effect of STAT3 inhibitor and PEDF receptor siRNA on hepatocyte apoptosis induced by serum deprivation. (A) Inhibitor of STAT3 prevents the induction of PEDF by the 44-mer. Hepatocytes were pretreated with STAT3 inhibitor or ERK inhibitor (PD98059) or PPARγ antagonist (GW9662) for 2 h and then treated with the 44-mer for 24 h. To examine the role of PEDF receptor on antiapoptotic effect of the 44-mer, hepatocytes were transfected with the indicated siRNA. Two days later, the hepatocytes were starved of serum and exposed to the 44-mer for further 24 h. Subsequently, apoptosis was determined by TUNEL assay. Graphs represent means ± SE (n = 4). *P<0.001 versus 44-mer-treated cell. **P < 0.05 relative to the cells pretreated with control siRNA/44-mer. (B) The 44-mer induces STAT3 phosphorylation. Hepatocytes were treated with the 44-mer for the indicated time periods and phosphorylated STAT3 were detected by western blot analysis. Representative immunoblots and densitometric analysis are shown as the mean ± SE (n = 3). *P < 0.001 versus untreated cells (time 0). (C-E) PNPLA2 siRNA reduces the PEDF/44-mer-induced STAT3 phosphorylation. Hepatocytes were transfected with control siRNA (lanes 2 and 3) or PEDF receptor specific siRNA (PNPLA2, LRP6 or LR; lanes 4 and 5) as indicated. Mock indicates cells treated with transfection reagent. PEDF receptor levels were normalized to the β-actin. Numbers below the blots refer to a densitometric measure expressed as a fold of mock control. To examine p-STAT3 levels, hepatocytes were treated with PEDF or 44-mer for 10 min and then lysed. Total and phosphorylated STAT3 protein levels were examined by western blotting. p-STAT3 was normalized to the STAT3. Graphs represent mean ± SE of 3 different experiments. *P < 0.05 versus Cont siRNA/PEDF-treated cells. **P < 0.05 versus Cont siRNA/44-mer-treated cells.

PEDF exerts multiple activities by activating several signaling pathways in different types of cells, such as the signal transducer and activator of transcription 3 (STAT3), ERK/mitogen-activated protein kinase (MAPK) and peroxisome proliferator-activated receptor gamma (PPARγ) [7,9,16]. Hence, we sought to determine whether these signaling pathways were also involved in the PEDF/44-mer mediated protective effect on hepatocytes. Fig 6A (left panels) shows that blockage of STAT3 activation by STAT3 inhibitor dose-dependently abolished the 44-mer inhibitory effect on serum deprivation-induced hepatocyte apoptosis. On the other hand, inhibitors of ERK and PPARγ had no apparent effect on hepatocyte apoptosis level induced by serum deprivation, suggesting that PEDF and the 44-mer protect hepatocytes from serum deprivation-induced apoptosis, possibly through a PPARγ/ERK-independent mechanism. In addition, Fig 6B shows that the 44-mer induced the STAT3 phosphorylation in rat hepatocytes, suggesting that the 44-mer activates STAT3 signal transduction pathway.

Cumulative evidence indicates that PEDF exerts its multiple functions by binding to various membrane receptors, including patatin-like phospholipase domain-containing protein 2/adipose triglyceride lipase (PNPLA2/ATGL), laminin receptor (LR) and Wnt co-receptor LDL receptor related protein 6 (LRP6) [27–29]. We investigated the involvement of PEDF receptors on the STAT3 phosphorylation induced by PEDF and the 44-mer. As shown in Fig 6C–6E, transfection of siRNA for PNPLA2, LR or LRP6, resulted in an 80% decrease in the PNPLA2, LR and LRP6 protein expression in comparison with control siRNA-treated cells and mock control, respectively. The PNPLA2 siRNA did not alter the expression of LRP6 and LR, which confirmed the specificity (S4 Fig). We determined whether PEDF receptor would affect the levels of p-STAT3 after stimulation by PEDF/44-mer for 10 min. As show in Fig 6C, PEDF and the 44-mer were unable to raise p-STAT3 levels in cells pretreated with PNPLA2 siRNA, compared with control siRNA, LR siRNA and LRP6 siRNA-transfected cells. The results suggest that PEDF and the 44-mer via interaction with PNPLA2 receptor to activate STAT3 signaling.

We also determined the effects of silencing PNPLA2 on the survival activity of the 44-mer. Primary rat hepatocytes pretreated with PNPLA2 siRNA significantly reduced the antiapoptotic activity of the 44-mer compared with cells pretreated with control siRNA, LR siRNA and LRP6 siRNA (13.7±0.8% versus 2.5±0.9%, 3.5±0.6% and 2.8±0.6%; Fig 6A, right panels), indicating that a dramatic decrease of PNPLA2 level in hepatocytes abolished PEDF’s capability to prevent hepatocyte apoptosis. Collectively, these suggest that PEDF and the 44-mer protect hepatocytes by binding to PNPLA2 receptor and activating STAT3 signaling.

### Loss of PEDF facilitates hepatocyte apoptosis

**Fig 7:**
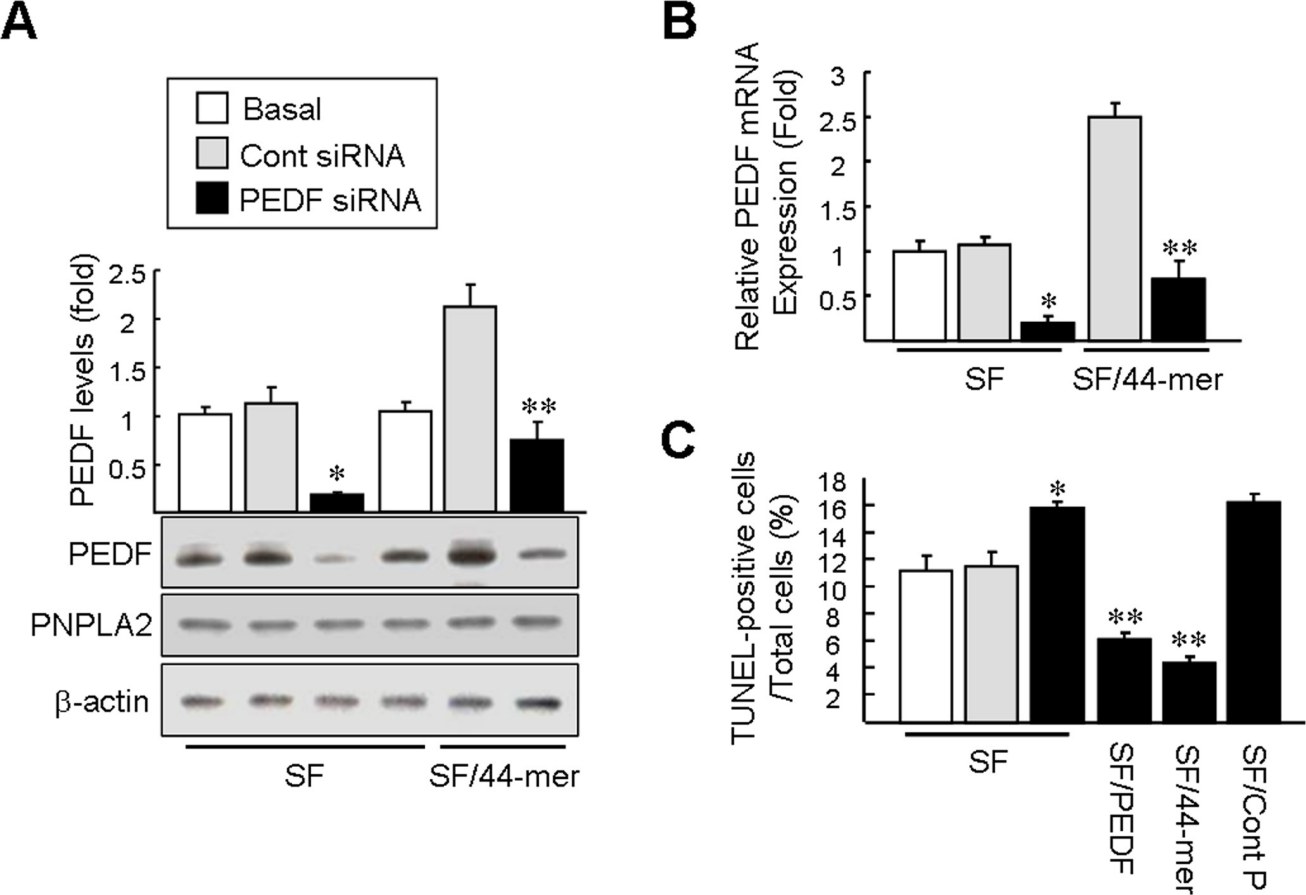
Knockdown of PEDF leads to a reduction in cell survival. Hepatocytes were transfected with control siRNA or PEDF specific siRNA as indicated. Two days later, the hepatocytes were serum starved for 24 h with or without 10 μM 44-mer. (A) Expression of the PEDF proteins was verified by Western blotting. PEDF levels were normalized to the β-actin. Graphs represent mean ± SE of 3 different experiments. *P< 0.002 versus SF/Cont siRNA-transfected cells. **P< 0.05 versus 44-mer/Cont siRNA-transfected cells. (B) Expression of the PEDF mRNA was verified by quantitative RT-PCR. The relative mRNA level was calculated from the mean value relative to the basal control. Results are means ± SE of triplicate transfections. *P< 0.001 versus SF/Cont siRNA-transfected cells. **P< 0.02 versus 44-mer/Cont siRNA-transfected cells. (C) TUNEL assay. The percentage of cell apoptosis was quantified by dividing the number of apoptotic nuclei to a population of 2000 counted cells per condition. Graphs represent means ± SE (n = 3). *P< 0.01 versus SF/Cont siRNA-transfected cells. **P< 0.001 relative to the Cont peptide/PEDF siRNA-transfected cells.

Hepatocytes produce PEDF in vivo and in cell culture. To evaluate the role of endogenous PEDF in cell survival, PEDF specific siRNAs targeting rat PEDF were transfected into primary rat hepatocytes. Western blot analysis confirmed that transfection of PEDF siRNA led to a pronounced decrease in the intracellular protein level by a factor of 6.2-fold, compared with transfection of control siRNA (left planes; Fig 7A). In addition, the PEDF siRNAs did not alter the expression of PNPLA2, which confirmed the specificity. Remarkably, even though the 44-mer stimulation increased endogenous PEDF protein levels by 2.1-fold as compared with cells incubated in SF medium (basal), knockdown of PEDF could block the increased protein expression of endogenous PEDF induced by the 44-mer (right planes; Fig 7A). In addition, the rat PEDF siRNA suppressed the PEDF mRNA steady-state levels and blocked the ability of the 44-mer to induce endogenous PEDF mRNA expression (Fig 7B). The effect of PEDF knockdown on the response to serum deprivation was analyzed by a TUNEL assay. Cells with low levels of endogenous PEDF led to an increase in TUNEL-positive cells compared with SF/control siRNA (Fig 7C; left planes; 15.9±0.5% versus 11.5± 1.1%), strongly indicating that endogenous PEDF plays an important role in the cell protection. However, the inhibitory effect of PEDF siRNA on cell survival was completely blocked by addition of exogenous PEDF and the 44-mer, compared with addition of control peptide (6.2±0.5% and 4.4±0.5% versus 16.3± 0.6%). Collectively, the result demonstrated that hepatocytes were more sensitive to the serum deprivation-induced cell apoptosis if PEDF levels were low.

### The 44-mer prevents CCl_4_-induced hepatic fibrosis in a model of chronic liver damage

**Fig 8:**
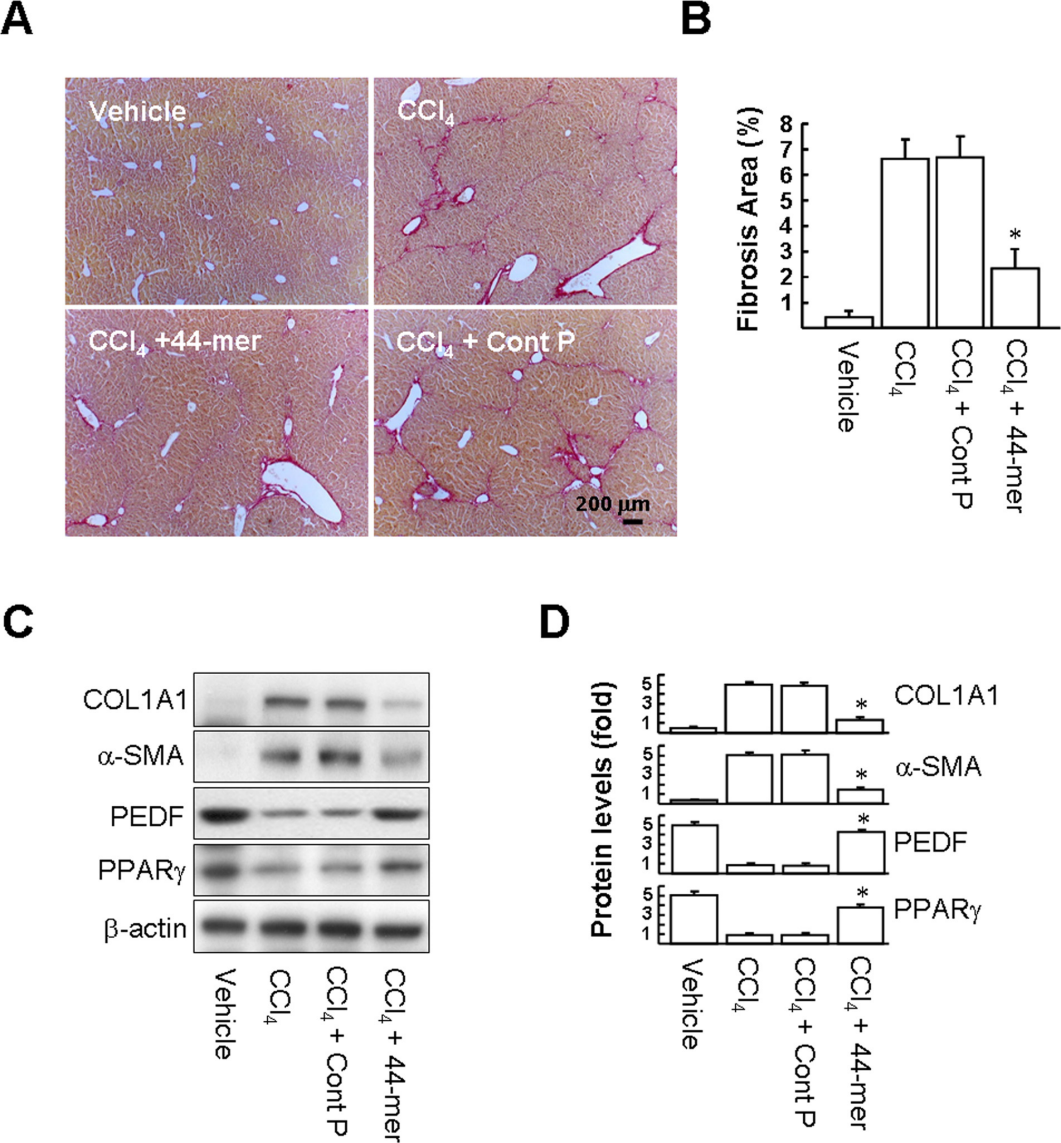
The 44-mer protects mice from CCl_4_-induced hepatic fibrosis. Mice were treated with CCl_4_ twice per week for 5 weeks. (A) Histopathological detection of collagen in the liver by Sirius red-staining. (Original magnification, ×200). Representative images from five mice in each subgroup are shown. (B) Estimation of the area of hepatic fibrosis by Sirius red staining. Data were assessed by analyzing 10 Sirius red-stained liver sections per animal with a computerized image system. *P <0.001 *versus* the CCl_4_+control peptide-treated group. (C and D) Whole liver protein lysates at week 5 post-CCl_4_ treatment were extracted for western blot analysis with the antibodies indicated. Representative blots and densitometric analysis are shown from five mice in each subgroup. *P <0.005 *versus* CCl_4_+control peptide-treated group. **P <0.01 *versus* CCl_4_+control peptide-treated group.

To investigate the protective effect of the 44-mer in liver injury induced by repeated administration of CCl_4_, mice were given intraperitoneal CCl_4_ injections twice per week for 5 weeks. The extent of liver fibrosis was investigated by Sirius red staining. As depicted in Fig 8A, liver sections from CCl_4_-treated mice showed marked parenchymal injury and bridging fibrosis. The 44-mer treatment reduced parenchymal injury and smaller areas of fibrosis were observed, compared to those seen in the CCl_4_ and CCl_4_/Cont P groups (Fig 8B; 2.3 ± 0.78% versus 6.6 ± 0.76% and 6.7± 0.85%, respectively). We also evaluated the levels of α-SMA, COL1A1, PEDF and PPARγ in liver protein extracts harvested at week 5 (Fig 8C), the levels of COL1A1 and α-SMA were reduced by factors of 3.1-fold and 2.5-fold, respectively, in the CCl_4_/44-mer treated group compared to the CCl_4_/control peptide group (Fig 8D). The reduced α-SMA and COL1A1 protein levels seen in the 44-mer-treated mice were correlated with a lower level of collagen deposition, suggesting that the development of fibrosis is suppressed by the 44-mer administration. Hepatic PEDF and PPARγ are intrinsic protectors against liver cirrhosis [7]. The CCl_4_/44-mer treated group also showed a significant increase in PEDF and PPARγ protein levels, by 5.1-fold and 4.1-fold, respectively, compared to CCl_4_/Cont P group. Collectively, the protective effect of the 44-mer was also reproducible in CCl_4_-induced chronic liver injury in mice.

## Discussion

PEDF is strongly expressed in the liver and has antifibrotic properties [7]. The mechanism through which PEDF protects against liver fibrosis is being elucidated gradually. A 34-mer peptide derived from PEDF was found to alleviate CCl_4_-induced liver fibrosis by suppressing the fibrogenic responses of rat hepatic stellate cells (HSCs) and through inhibiting platelet-derived growth factor (PDGF)-mediated mitotic signaling [9]. This suggests that prevention of HSC activation is the basis of the anti-fibrotic mechanism of PEDF. However, because inflammation induced by repeated hepatocyte injury is the major cause of liver fibrosis [18,30], PEDF could also prevent liver fibrosis by directly protecting liver cells from damage. This possibility is supported by data provided in this study; our animal experiments show that a 44-mer peptide protects hepatocytes against CCl_4_ damage. Consistent with the in vivo results, PEDF and the 44-mer can block the apoptosis of primary culture of rat hepatocytes induced by serum deprivation and TGF-β1. Our study, therefore, provides the first evidence that PEDF can prevent liver fibrosis by direct protection of hepatocytes and this protective effect is manifested through its 44-mer domain.

The cytochrome P_450_ oxygenase system metabolizes CCl_4_ into free radicals, such as trichloromethyl peroxide (CCl_3_) and trichloroperoxyl radical (CCl_3_O_2_^-^). Both free radicals lead to lipid peroxidation and injure the hepatocytes. Other chemicals that damage liver cells, such as ethanol, also generate free oxygen radicals. Many liver protectants have been reported to have antioxidant properties or to scavenge ROSs [31–33]. Enhancement of free radical handling by PEDF has been reported; PEDF and the 44-mer protect cardiomyocytes from oxidative stress induced by hypoxia via up-regulation of SOD, catalase and GPx levels [14]. All of these enzymes have been reported to protect liver cells against CCl_4_-mediated oxidative damage [31,33]. In addition, PEDF increases the levels of reduced GSH to protect endothelial cells against damage from oxidative stress induced by high glucose concentrations [34]. It has been shown that the extent of liver GSH depletion is closely correlated with lipid peroxidation in CCl_4_-induced acute hepatic damage in mice [35]. Our present study supports the concept that the 44-mer administration rescued GSH levels, concomitant with decreased MDA formation. We also investigated the enzymes relative to the GSH redox system. A significant decrease in hepatic GPx and GR activity was found in mice treated with CCl_4_ alone. In contrast, in mice also treated with the 44-mer, the hepatic GPx and GR activities were suppressed less by CCl_4_. These results suggest that the 44-mer exerted a beneficial effect on hepatic GSH restoration in this animal model.

There are multiple mechanisms of CCl_4_ hepatotoxicity. Initially, highly reactive free radicals are formed through the metabolism of CCl_4_, which then causes lipid peroxidation, thereby leading to liver necrosis. Later, the damaged hepatocytes release free radicals and apoptotic bodies that cause activation of Kupffer cells/macrophages, which results in the production of pro-inflammatory mediators, including TNF-α and other pro-inflammatory cytokines [18,36,37]. Persistent accumulation of TNF-α has been shown to enhance the hepatocyte damage further [19,20]. In this study, the 44-mer treatment suppressed serum and hepatic TNF-α and IL-1β expression induced by CCl_4_ injection. The 44-mer effect may be associated with its hepatocellular protection or regulation of the inflammatory responses of Kupffer cells. In searching for the hepatoprotective mechanism of the 44-mer, we found that the levels of hepatic Bcl-xL and PEDF were suppressed less by CCl_4_ when the mice were also administered the 44-mer. In contrast, the 44-mer suppressed CCl_4_-induced Bax and activated caspase-3 proteins. In this study, we also observed that the 44-mer induces activated STAT3 (p-STAT3) in the livers of CCl_4_-treated mice and primary culture of rat hepatocytes. Previous studies have proved that Bcl-xL, PEDF and STAT3 are hepatoprotective factors. For example, Bcl-xL plays a critical role in fibronectin and pregnane X receptor (PXR)-mediated antiapoptotic signaling of hepatocytes after acute liver injury [38,39]. In addition, PEDF-null mice suffer from spontaneous liver fibrosis [40]. Meanwhile, a previous study has shown that expression of Bcl-xL mRNA in hepatoma HepG2 cells is decreased significantly by a small interfering RNA targeting PEDF, implying that PEDF can up-regulate *bcl-xL* gene expression [41]. Therefore, the hepatoprotective effect of the 44-mer may be associated with the restoration of hepatic PEDF and Bcl-xL in this animal model. Activation of STAT3 in hepatocytes and Kupffer cells has been shown to ameliorate hepatocellular necrosis and inflammatory cytokine production, respectively [42]. The anti-inflammatory effects of IL-10 in the liver are likely mediated via activation of STAT3 in Kupffer cells [43]. Our previous study demonstrated that the 44-mer induces STAT3 phosphorylation in limbal stem cells and myoblasts [16,44]. Here we further found that PEDF and the 44-mer induce STAT3 phosphorylation via PNPLA2 receptor in primary rat hepatocytes and that this pathway is essential for PEDF/44-mer-mediated antiapopototic action. Further studies may be needed to elucidate whether the 44-mer can activate STAT3 in Kupffer cells and the signaling effect on inflammatory responses.

In the present study, we demonstrated that a small peptide fragment of the human PEDF molecule could display the hepatoprotective action and that the protective activity is likely to be mediated directly via a single class of PEDF receptors. It has been suggested strongly that the ligand binding domain (LBD) of PNPLA2 is able to interact with the PEDF 44-mer [45]. PNPLA2/ATGL seems to be the receptor most likely mediating liver cell protection following stimulation by the 44-mer. PEDF can active the PNPLA2-NF-κB pathway to protect retinal ganglion cells (RGC) from apoptosis induced by hypoxia [46]. Moreover, it has been shown that ATGL protects adipocytes against oxidative stress-induced cell death by promoting the expression of genes associated with mitochondrial oxidative metabolism [47]. In addition, PNPLA2/ATGL and PEDF are co-expressed in hepatocytes in response to the effects of triglyceride metabolism [48]. On the other hand, the laminin receptor is responsible for the anti-angiogenic activity of PEDF and the 34-mer through its mediation of endothelial cell apoptosis [27]. As for LRP6, the 34-mer, rather than the 44-mer, has been shown to bind to it and suppress the fibrogenic activation of HSCs [9]. Previously, we reported that mice administered CCl_4_ show a dramatic reduction of hepatic PEDF, whereas AAV-mediated gene delivery of human PEDF is capable of halting the progression of liver fibrosis in experimental animals [7]. In addition, the levels of murine hepatic PEDF were suppressed less by CCl_4_ [7]. However, in the current study, we show that exogenous PEDF/44-mer can trigger sufficient cell protection in PEDF knockdown hepatocytes cultured in serum deprivation condition. Nonetheless, the 44-mer mediates PEDF expression in vivo that may still provide survival advantage by the 44-mer independent but PEDF-dependent activities, such as suppression of the platelet-derived growth factor (PDGF)-mediated HSC activation during liver fibrogenesis [9].

Overall, this study is the first to show that a synthetic 44-mer peptide has protective effects against CCl_4_-induced acute hepatic injury and liver fibrosis. The protective mechanism might involve inhibition of oxidative stress, inflammatory responses and apoptosis, as well as the induction of hepatoprotective factors. The precise molecular mechanism that mediates the cellular antioxidative system requires further investigation.

## Supporting Information

S1 Fig The 44-mer and control peptide effect on plasma ALT/AST levels induced by CCl_4_. C57BL/6 mice (three mice per experimental condition) by a single intraperitoneal injection of CCl4 solution (5 ml/kg body weight, as a 1:4 mixture with olive oil) and treated with varying doses of the 44-mer or control peptide for 24 h, 48 h and 72 h.
(DOC)

S2 Fig The 44-mer partially sustains hepatic PPARγ protein levels in mice at 48 h after a single injection of CCl_4_. Liver protein extracts were harvested and subjected to western blot analysis with antibodies as indicated. Representative blots and densitometric analysis from 3 independent experiments are shown. The immunoblots were scanned and quantitated at individual sites and normalized to β-actin. *P <0.05 versus CCl_4_+control peptide-treated group.
(DOC)

S3 Fig Effect of PEDF and the 44-mer on serum deprivation-induced cell apoptosis. Primary rat hepatocytes were either left cultured in serum-free (SF) medium or SF medium supplemented with different doses of PEDF or 44-mer for 24 h. Apoptosis was determined by TUNEL staining and doubly stained with Hoechst 33258. The percentage of cell death was quantified by dividing the number of TUNEL-positive cells to a population of 2000 counted cells per condition. Graphs represent means ± SE (n = 4). *P<0.001 versus cell treated with solvent.
(DOC)

S4 Fig Effect of PNPLA2 siRNA on the protein levels of PNPLA2, LRP6 and LR in rat primary hepatocytes. Hepatocytes were transfected with control siRNA or PNPLA2 specific siRNA as indicated. Mock indicates cells treated with transfection reagent. At 24 h after siRNA transfection, hepatocytes were resuspended in new culture media for recovery for 24 h and then subjected to western blot analysis with antibodies as indicated. Graphs represent three independent experiments.
(DOC)

S1 Table Primers used in the quantitative real-time RT-PCR. (DOC)

S2 Table siRNA sequences used in the experiment. (DOC)
