# Supplementary material for: Pigment Epithelium Derived Factor Peptide Protects Murine Hepatocytes from Carbon Tetrachloride-Induced Injury
Source: PLoS One. 2016 Jul 6;11(7):e0157647. doi: 10.1371/journal.pone.0157647 (PMC4934881; doi:10.1371/journal.pone.0157647)
Supplement: S1 Fig — C57BL/6 mice (three mice per experimental condition) by a single intraperitoneal injection of CCl4 solution (5 ml/kg body weight, as a 1:4 mixture with olive oil) and treated with varying doses of the 44-mer or control peptide for 24 h, 48 h and 72 h. (DOC) [file pone.0157647.s001.doc]

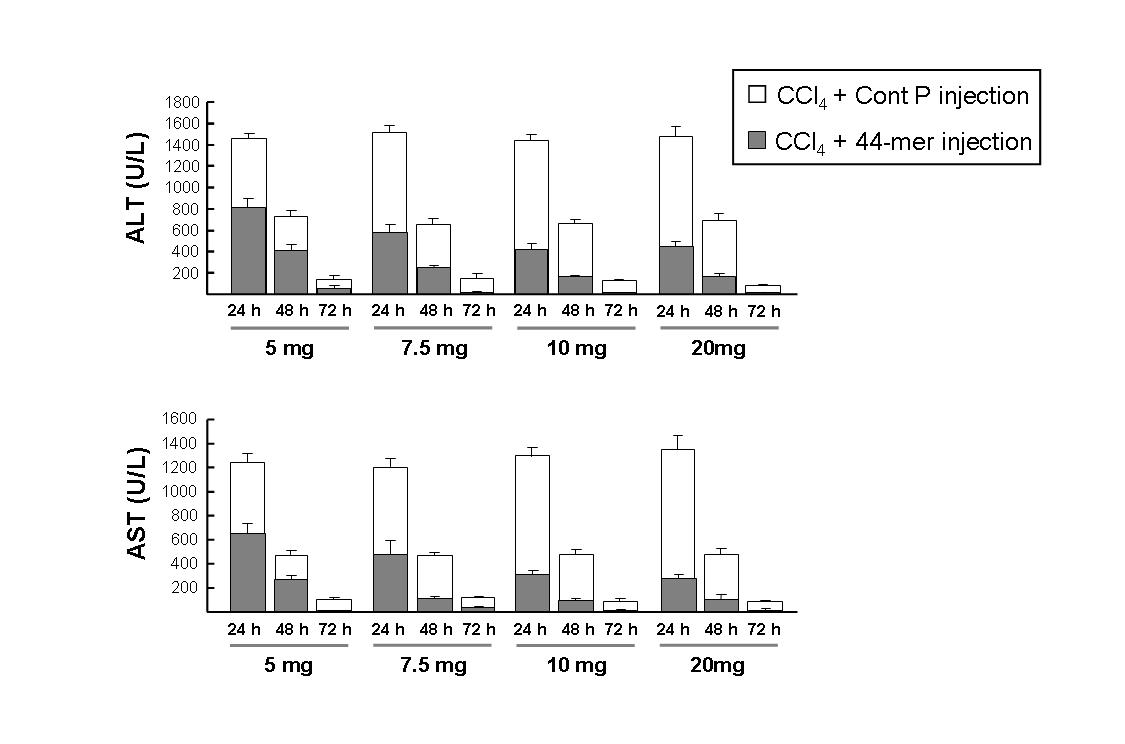


**S1 Fig. The 44-mer and control peptide effect on plasma ALT/AST levels induced by CCl_4_.** C57BL/6 mice (three mice per experimental condition) by a single intraperitoneal injection of CCl4 solution (5 ml/kg body weight, as a 1:4 mixture with olive oil) and treated with varying doses of the 44-mer or control peptide for 24 h, 48 h and 72 h.
