# Supplementary material for: Pigment Epithelium Derived Factor Peptide Protects Murine Hepatocytes from Carbon Tetrachloride-Induced Injury
Source: PLoS One. 2016 Jul 6;11(7):e0157647. doi: 10.1371/journal.pone.0157647 (PMC4934881; doi:10.1371/journal.pone.0157647)
Supplement: S2 Fig — Liver protein extracts were harvested and subjected to western blot analysis with antibodies as indicated. Representative blots and densitometric analysis from 3 independent experiments are shown. The immunoblots were scanned and quantitated at individual sites and normalized to β-actin. *P <0.05 versus CCl4+control peptide-treated group. (DOC) [file pone.0157647.s002.doc]

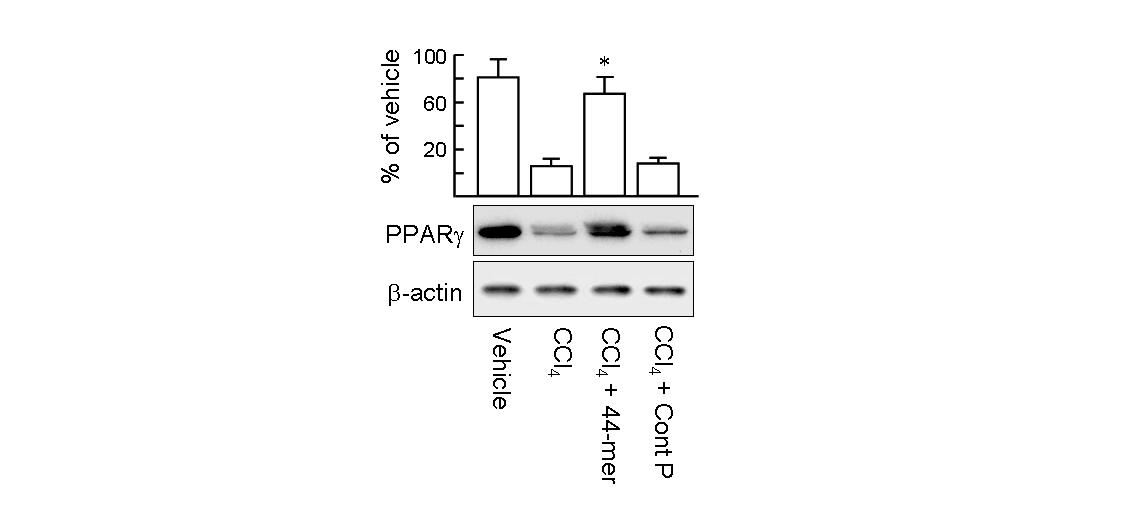


**S2 Fig. The 44-mer partially sustains hepatic PPARγ protein levels in mice at 48 h after a single injection of CCl_4_.** Liver protein extracts were harvested and subjected to western blot analysis with antibodies as indicated. Representative blots and densitometric analysis from 3 independent experiments are shown. The immunoblots were scanned and quantitated at individual sites and normalized to β-actin. **P* <0.05 versus CCl_4_+control peptide-treated group.
