# Supplementary material for: Pigment Epithelium Derived Factor Peptide Protects Murine Hepatocytes from Carbon Tetrachloride-Induced Injury
Source: PLoS One. 2016 Jul 6;11(7):e0157647. doi: 10.1371/journal.pone.0157647 (PMC4934881; doi:10.1371/journal.pone.0157647)
Supplement: S3 Fig — Primary rat hepatocytes were either left cultured in serum-free (SF) medium or SF medium supplemented with different doses of PEDF or 44-mer for 24 h. Apoptosis was determined by TUNEL staining and doubly stained with Hoechst 33258. The percentage of cell death was quantified by dividing the number of TUNEL-positive cells to a population of 2000 counted cells per condition. Graphs represent means ± SE (n = 4). *P<0.001 versus cell treated with solvent. (DOC) [file pone.0157647.s003.doc]

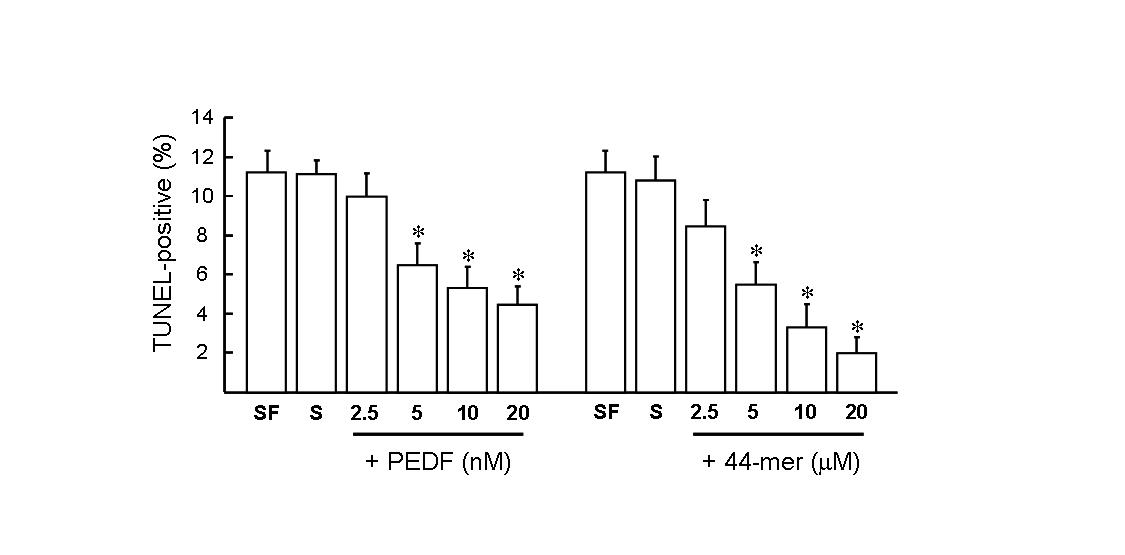


**S3 Fig. Effect of PEDF and the 44-mer on serum deprivation-induced cell apoptosis.** Primary rat hepatocytes were either left cultured in serum-free (SF) medium or SF medium supplemented with different doses of PEDF or 44-mer for 24 h. Apoptosis was determined by TUNEL staining and doubly stained with Hoechst 33258. The percentage of cell death was quantified by dividing the number of TUNEL-positive cells to a population of 2000 counted cells per condition. Graphs represent means ± SE (n=4). **P*<0.001 versus cell treated with solvent.
