# Supplementary material for: Pigment Epithelium Derived Factor Peptide Protects Murine Hepatocytes from Carbon Tetrachloride-Induced Injury
Source: PLoS One. 2016 Jul 6;11(7):e0157647. doi: 10.1371/journal.pone.0157647 (PMC4934881; doi:10.1371/journal.pone.0157647)
Supplement: S4 Fig — Hepatocytes were transfected with control siRNA or PNPLA2 specific siRNA as indicated. Mock indicates cells treated with transfection reagent. At 24 h after siRNA transfection, hepatocytes were resuspended in new culture media for recovery for 24 h and then subjected to western blot analysis with antibodies as indicated. Graphs represent three independent experiments. (DOC) [file pone.0157647.s004.doc]

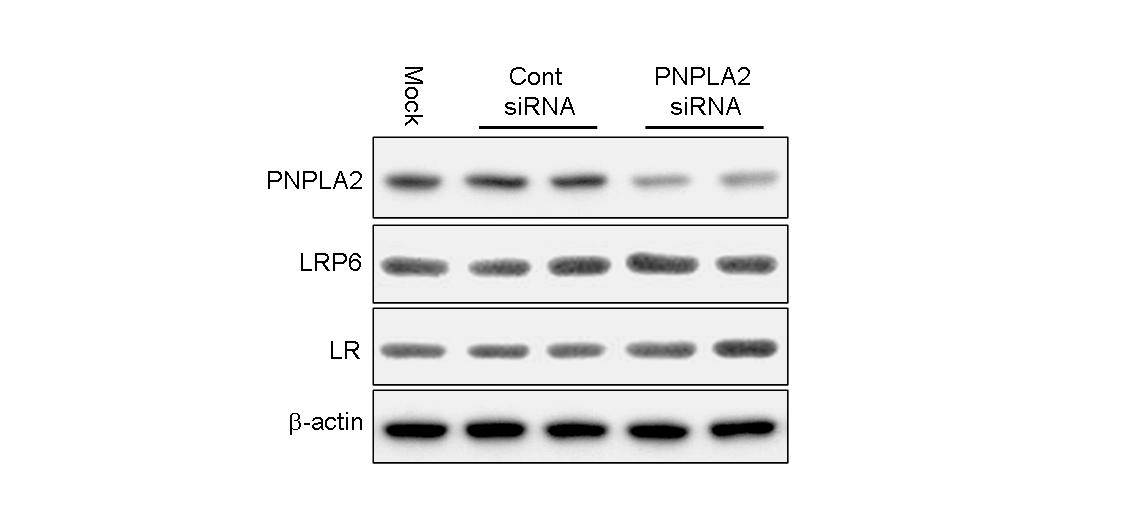


**S4 Fig. Effect of PNPLA2 siRNA on the protein levels of PNPLA2, LRP6 and LR in rat primary hepatocytes.** Hepatocytes were transfected with control siRNA or PNPLA2 specific siRNA as indicated. Mock indicates cells treated with transfection reagent. At 24 h after siRNA transfection, hepatocytes were resuspended in new culture media for recovery for 24 h and then subjected to western blot analysis with antibodies as indicated. Graphs represent three independent experiments.
