# Supplementary figures and images for: Pigment Epithelium Derived Factor Peptide Protects Murine Hepatocytes from Carbon Tetrachloride-Induced Injury
Source: PLoS One. 2016 Jul 6;11(7):e0157647. doi: 10.1371/journal.pone.0157647 (PMC4934881; doi:10.1371/journal.pone.0157647)

**S1 Table.** Primers used in the quantitative real-time RT-PCR


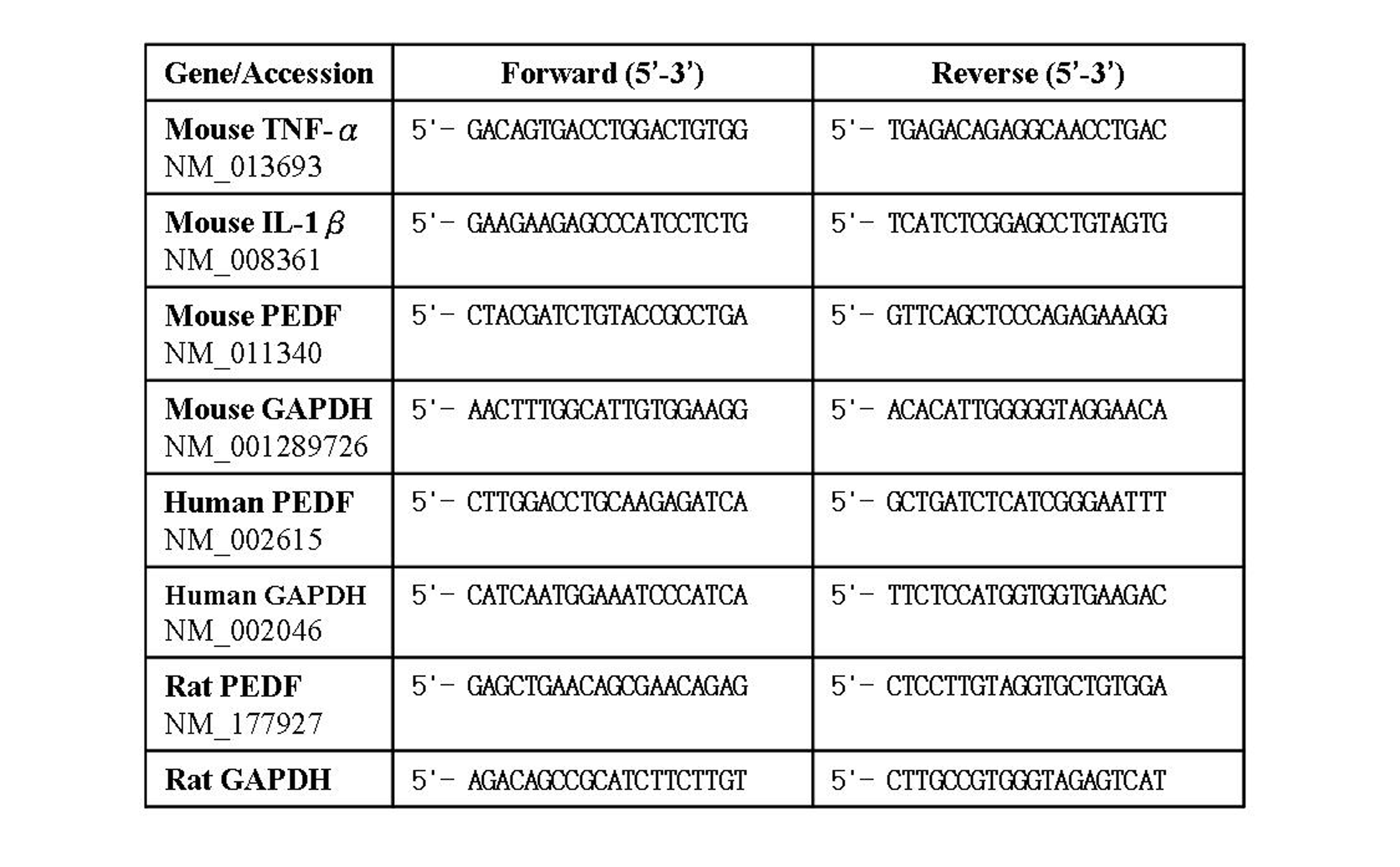

Supplement: S1 Table — (DOC) [file pone.0157647.s005.doc]

**S2 Table.** siRNA sequences used in the experiment


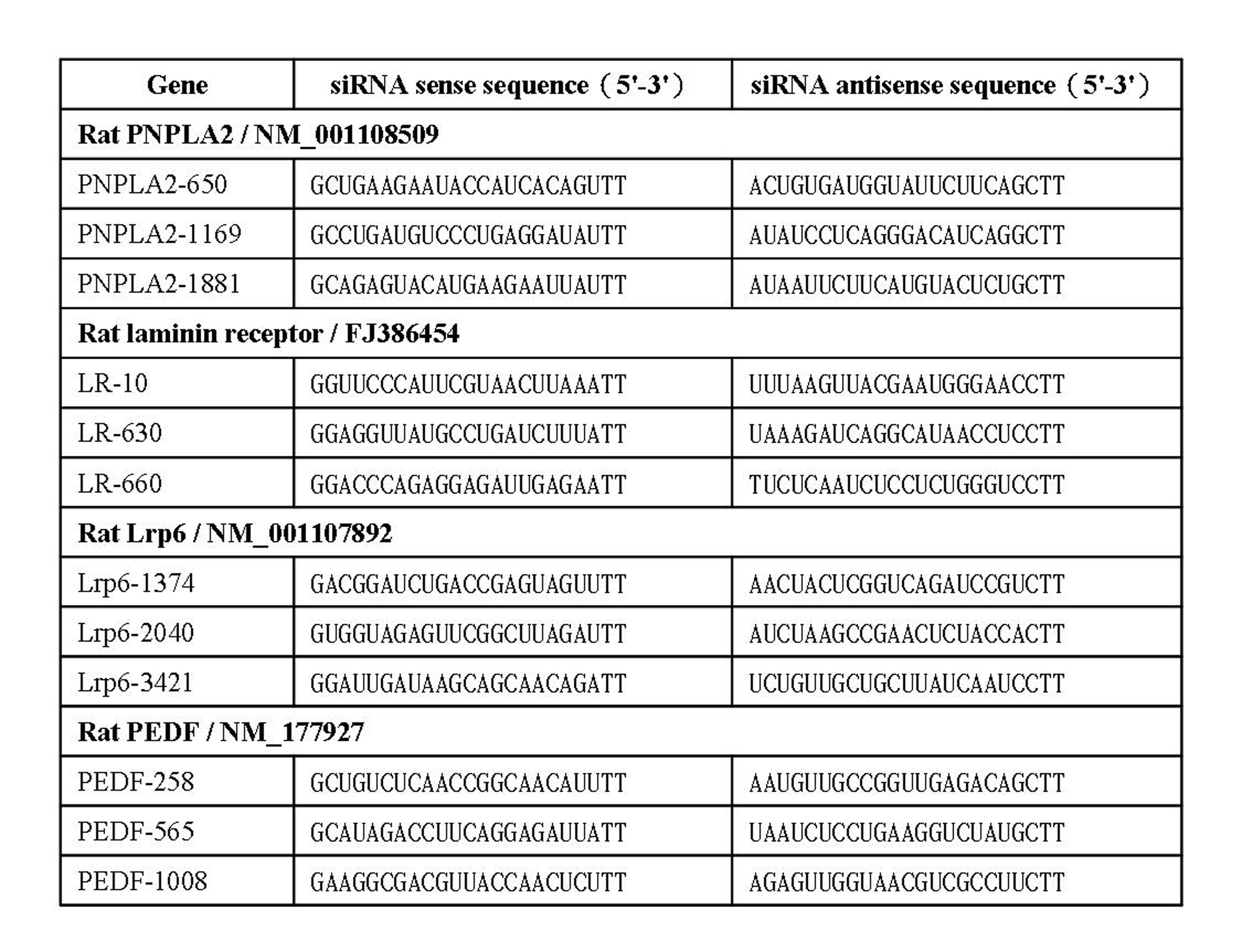

Supplement: S2 Table — (DOC) [file pone.0157647.s006.doc]
